# Full restoration of specific infectivity and strain properties from pure mammalian prion protein

**DOI:** 10.1371/journal.ppat.1007662

**Published:** 2019-03-25

**Authors:** Cassandra M. Burke, Daniel J. Walsh, Alexander D. Steele, Umberto Agrimi, Michele Angelo Di Bari, Joel C. Watts, Surachai Supattapone

**Affiliations:** 1 Departments of Biochemistry and Cell Biology at Darthmouth, Hanover, New Hampshire, United States of America; 2 Department of Veterinary Public Health and Food Safety, Istituto Superiore di Sanità, Rome, Italy; 3 Tanz Centre for Research in Neurodegenerative Diseases and Department of Biochemistry, University of Toronto, Toronto, Ontario, Canada; 4 Geisel School of Medicine at Dartmouth, Hanover, New Hampshire, United States of America; University of Alberta, CANADA

## Abstract

The protein-only hypothesis predicts that infectious mammalian prions are composed solely of PrP^Sc^, a misfolded conformer of the normal prion protein, PrP^C^. However, protein-only PrP^Sc^ preparations lack significant levels of prion infectivity, leading to the alternative hypothesis that cofactor molecules are required to form infectious prions. Here, we show that prions with parental strain properties and full specific infectivity can be restored from protein-only PrP^Sc^ *in vitro*. The restoration reaction is rapid, potent, and requires bank vole PrP^C^ substrate, post-translational modifications, and cofactor molecules. To our knowledge, this represents the first report in which the essential properties of an infectious mammalian prion have been restored from pure PrP without adaptation. These findings provide evidence for a unified hypothesis of prion infectivity in which the global structure of protein-only PrP^Sc^ accurately stores latent infectious and strain information, but cofactor molecules control a reversible switch that unmasks biological infectivity.

## Author summary

Prions are unusual infectious agents that cause invariably fatal brain diseases. Unlike conventional infectious agents such as bacteria or viruses, prions do not possess nucleic acids such as DNA or RNA, and therefore it is not clear how they are able to replicate and cause infection. A leading model is that prions are composed exclusively of a specific protein molecule with an abnormal shape, which has the ability to coerce other protein molecules to change into the same abnormal shape in a self-reinforcing process. Although this model is attractive, no one has ever been able to make potently infectious prions from only pure protein. Here, we show for the first time that pure protein can faithfully store and transmit specific infectious information (strain properties) in a latent state even though it is non-infectious.

## Introduction

Prion diseases are a class of infectious, invariably fatal neurodegenerative diseases that affect humans and other mammals. Examples of prion diseases include Creutzfeldt-Jakob disease (CJD) in human patients, chronic wasting disease (CWD) in cervids including deer and elk, bovine spongiform encephalopathy (BSE) in cattle, and scrapie in sheep and goats [1]. A key pathogenic event in prion diseases is the conversion of the host-encoded prion protein from its normal, cellular conformation—termed PrP^C^—into a self-replicating, misfolded conformation—termed PrP^Sc^—which is typically protease-resistant.

The protein-only hypothesis posits that infectious mammalian prions are composed solely of PrP^Sc^ [1, 2]. Pure self-replicating protein conformers have been directly shown to mediate efficient and faithful inheritance of biological traits and strain properties in fungi[3–5]. However, no similar experimental evidence has been obtained to support the protein-only hypothesis for mammalian prions[6]. Amyloid fibrils containing only wild-type recombinant (rec) PrP can induce prion disease in transgenic mice [7], and induce prion formation by passage in asymptomatic wild-type mice [8] and hamsters [9]. Additionally, infectious amyloids have been generated using a disease-linked PrP truncation mutant [10]. Also, seeded propagation of recPrP without cofactors can produce prions with low levels of specific infectivity [11, 12]. However, in each of these cases, very large quantities of pure PrP were required to induce disease, often with long incubation times and incomplete attack rates in normal hosts. In other cases, it has been shown that high concentrations of pure PrP amyloid fibrils can eventually induce the formation of prions with unusual strain characteristics after a slow *in vivo* adaptation process in asymptomatic animals[8, 9]. To our knowledge, wild-type prions with significant levels of specific infectivity and faithful maintenance of parental strain properties have never been produced directly from PrP alone, raising the possibility that factors other than pure PrP may be necessary for efficient, high-fidelity replication of fully infectious prions [6].

Building upon the discovery of the membrane phospholipid phosphatidylethanolamine as an endogenous cofactor for mouse prion formation [13], our laboratory used the serial protein misfolding cyclic amplification (sPMCA) technique developed by Soto and colleagues [14, 15] to generate two self-replicating recombinant (rec) mouse (Mo) PrP^Sc^ conformers derived from the same original infectious template. The only difference between the two conformers is that one sample was produced with a substrate cocktail containing recPrP plus purified phospholipids (Mo cofactor recPrP^Sc^), while the other was produced with recPrP alone (Mo protein-only recPrP^Sc^)[16]. These two autocatalytic conformers share a similar global structure but display strikingly different levels of specific infectivity in mice [16, 17]. Based on end-point titration bioassays, the difference in specific infectivity between Mo cofactor recPrP^Sc^ and Mo protein-only recPrP^Sc^ in wild-type mice is >10^5^ fold, with Mo protein-only recPrP^Sc^ causing no disease at all. The inability of Mo protein-only recPrP^Sc^ to infect WT mice can be explained by its inability to seed native Mo PrP^C^ substrate in brain homogenate (BH) sPMCA, whereas Mo cofactor recPrP^Sc^ effectively converts native MoPrP^C^ into PrP^Sc^ under the same conditions [16]. However, it is unknown whether a different host might be more receptive than mice to infection by Mo protein-only recPrP^Sc^.

Over the past decade, the European bank vole has emerged as an exciting model organism for prion disease research. Most animal species have transmission barriers that render them resistant to the majority of prion strains from other species. For example, humans appear to be susceptible to CJD and BSE, but not to CWD or scrapie [18–20], while dogs appear to be resistant to nearly all naturally occurring prion strains [21]. In contrast, the bank vole (*Myodes glareolus*) appears to be uniquely susceptible to nearly all prion strains from other animal species, except BSE [22–26]. This enhanced susceptibility can be directly attributed to the bank vole (BV) PrP^C^ sequence, because transgenic mice expressing BV PrP^C^ rather than Mo PrP^C^ are also near-universal hosts [25, 27].

We initially sought to determine whether bank voles might be more susceptible than mice to infection by protein-only recPrP^Sc^. This line of investigation led us to a series of unexpected results, which show that PrP^Sc^ alone can encode and propagate infectious information in a latent state, but that cofactor molecules are required to unmask biological infectivity.

## Results

### Bank vole brain homogenate is susceptible to seeding by protein-only recPrP^Sc^

**Fig 1:**
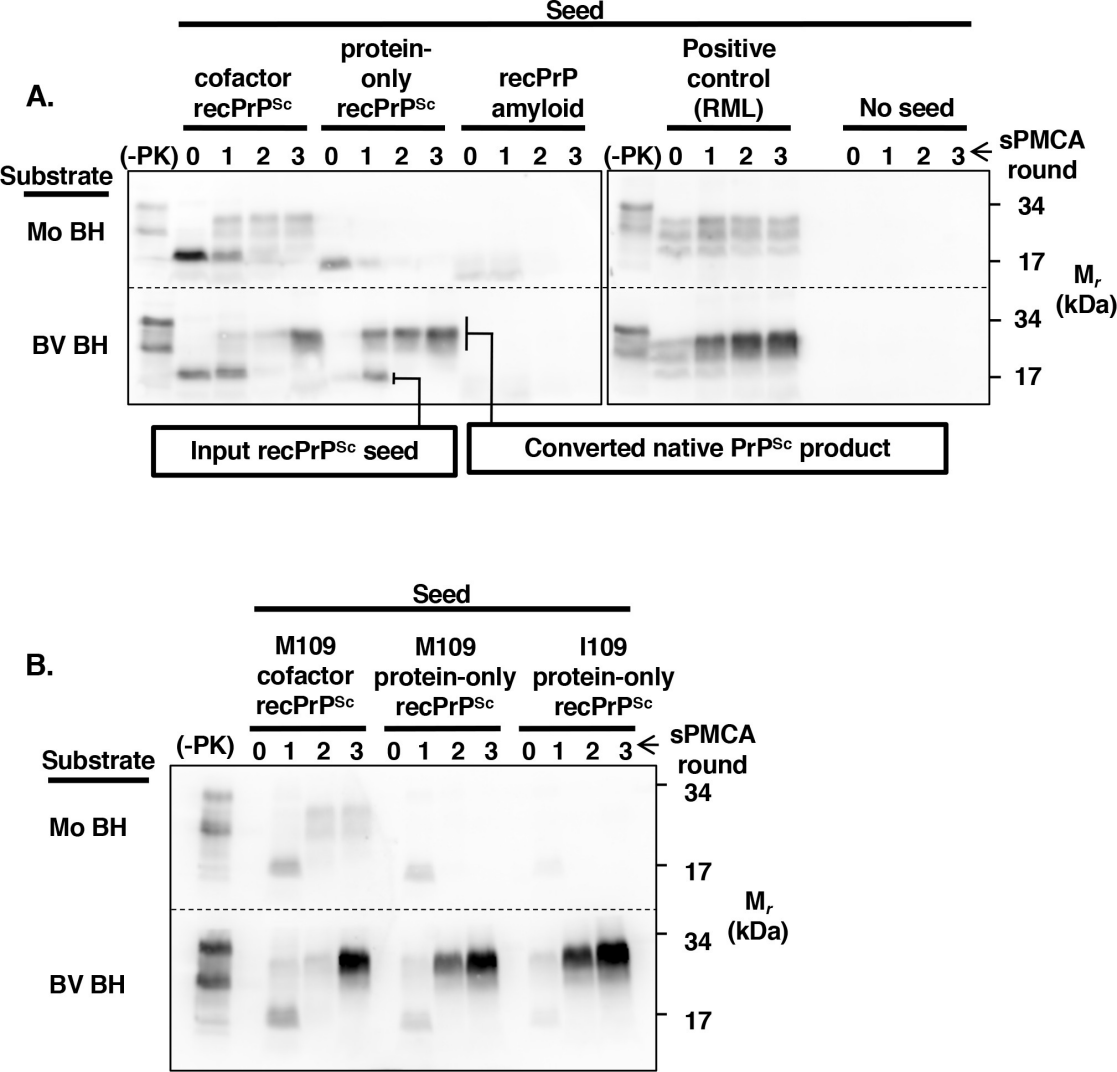
Comparison of the susceptibility of Mo BH and BV BH to different PrP^Sc^ seeds. Western blots probed with anti-PrP mAb 27/33 (epitope = 136–158 mouse numbering) showing three-round BH sPMCA reactions. Within each blot, reactions using Mo BH substrate are shown in the top row, and BV BH substrate in the bottom row. Reactions were seeded with various **(A)** Mo or **(B)** BV seeds, as indicated. The input seed concentration of all recPrP^Sc^ seeds was 6 μg/mL for a final reaction concentration of 0.6 μg/mL. Blots are representative of at least three independent experiments. −PK = sample not subjected to proteinase K digestion; all other samples were proteolyzed. Day 0 = seeded reaction not subjected to sonication. Note that input recPrP^Sc^ seeds migrate at a lower MW than the converted native PrP^Sc^ sPMCA product, as indicated by the boxed labels.

The sPMCA reactions and PrP^Sc^ conformers used in this paper are illustrated in **S1 Fig**. We first used BV BH sPMCA to assess the potential susceptibility of bank voles to protein-only recPrP^Sc^ [14]. As expected, self-propagating PrP^Sc^ molecules were successfully produced in both BV and Mo brain homogenates seeded by RML prions (**Fig 1A**, positive control), but not in unseeded reactions (**Fig 1A**, no seed), confirming that both homogenates are fundamentally competent substrates for sPMCA reactions. And, as previously reported, Mo brain homogenate could be seeded by Mo cofactor recPrP^Sc^, but not by Mo protein-only recPrP^Sc^ [28](**Fig 1A**, top row). Remarkably, we found that BV BH could be successfully seeded by Mo protein-only recPrP^Sc^ (**Fig 1A**, bottom row; note that newly-formed native BV PrP^Sc^ product migrates at ~27–30 kDa whereas Mo protein-only recPrP^Sc^ seed migrates at ~16 kDa). Moreover, a substantial amount of native PrP^Sc^ could be detected immediately during the first-round sPMCA (**Fig 1A**, bottom row; protein-only recPrP^Sc^, sPMCA round 1, indicating a rapid rate of PrP^Sc^ formation). Notably, the native BV PrP^Sc^ sPMCA product formed by protein-only recPrP^Sc^ seeding was identical in MW (~27–30 kDa) and glycoform profile (predominantly diglycosylated) as the sPMCA product seeded by cofactor recPrP^Sc^. To investigate the seed-specificity of this effect, we tested the ability of the same concentration of Mo recPrP amyloid (a different conformer of pure recPrP[29, 30]) to seed BV BH, and found that it was unable to induce PrP^Sc^ in either BV or Mo BH (**Fig 1A**, recPrP amyloid).

We also tested the ability of BV recPrP^Sc^ conformers (M109 cofactor recPrP^Sc^, M109 protein-only recPrP^Sc^, and I109 protein-only recPrP^Sc^) to seed BV BH sPMCA reactions. As expected, we found that BV M109 cofactor recPrP^Sc^ could effectively propagate in both Mo and BV BH substrates (**Fig 1B**, left-hand blocs). Additionally, both M109 protein-only recPrP^Sc^ and I109 protein-only recPrP^Sc^ could seed sPMCA reactions containing BV BH, but not Mo BH (**Fig 1B**, middle and right-hand blocs, compare bottom *vs*. top). Taken together, these results show that BV BH has a unique capacity for propagating protein-only recPrP^Sc^ seeds with various primary amino acid sequences.

### Bank vole brain homogenate is highly sensitive to seeding by protein-only recPrP^Sc^

**Fig 2:**
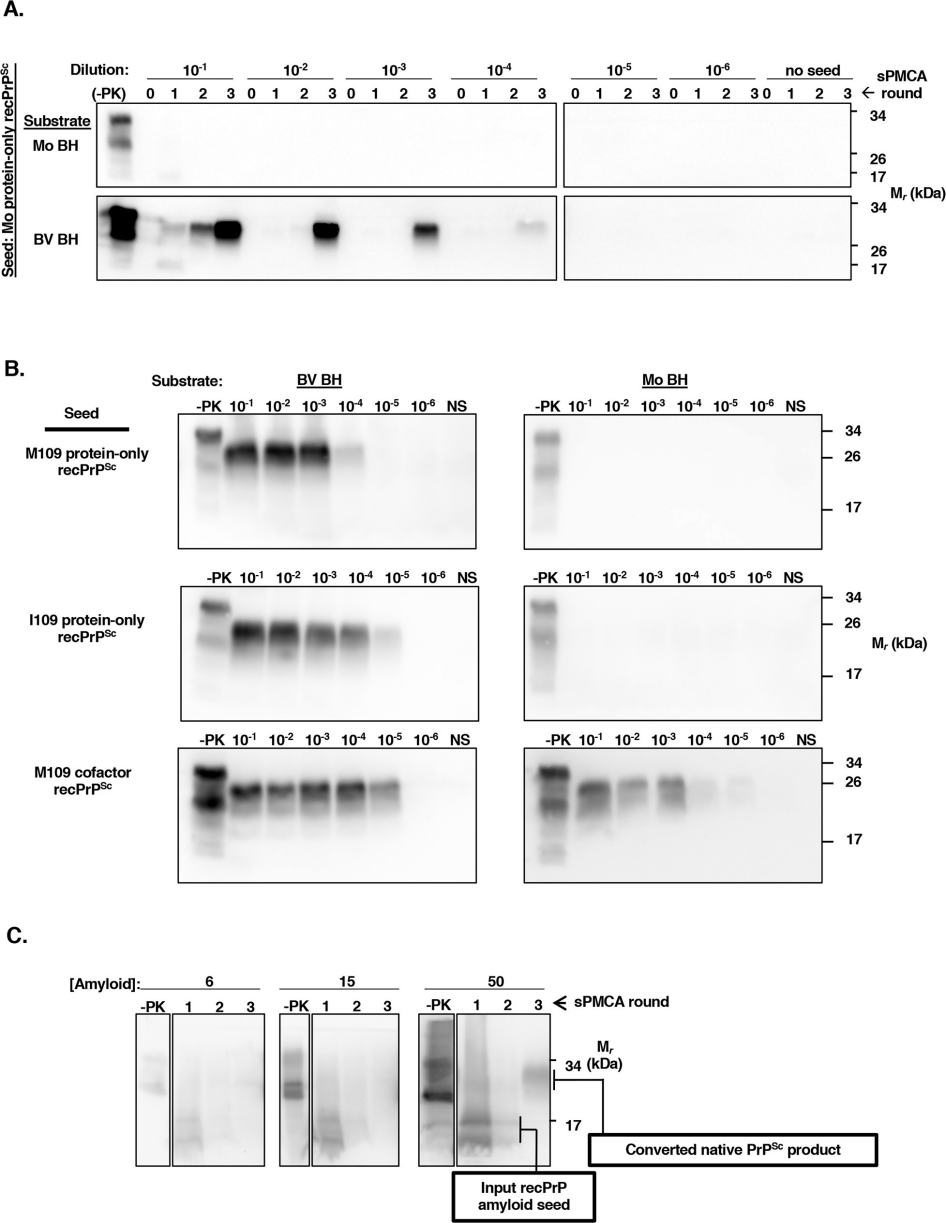
Protein-only recPrP^Sc^ seeds effectively propagate in BV BH, but not Mo BH sPMCA reactions. Western blots probed with anti-PrP mAb 27/33. **(A)** Titration of Mo protein-only recPrP^Sc^ *in vitro*. Western blots showing three-round BH sPMCA reactions with either Mo BH (top row) or BV BH (bottom row) substrates. Reactions were seeded with ten-fold serial dilutions of Mo protein-only recPrP^Sc^. The 10^−1^ reaction was seeded with 6 μg/mL of Mo protein-only recPrP^Sc^ for a final reaction concentration of 0.6 μg/mL of seed. **(B)** Titration of BV recPrP^Sc^ seeds *in vitro*. Western blots showing the third round of three-round BH sPMCA reactions with either BV BH (left column) or Mo BH (right column) substrates. Reactions were seeded with 10-fold serial dilutions of the indicated BV recPrP^Sc^ seed. The 10^−1^ reactions were seeded with 6 μg/mL of recPrP^Sc^ for a final reaction concentration of 0.6 μg/mL. NS- no seed. **(C)** M109 recPrP amyloid seeded three-round BV BH sPMCA reactions. Reactions were seeded with the increasing concentrations of amyloid, as indicated. Note that input recPrP amyloid seeds migrate at a lower MW than the converted native PrP^Sc^ sPMCA product, as indicated by the boxed labels.

To determine the seeding potency of protein-only recPrP^Sc^ seeds in BV BH, we tested serial 10-fold dilutions of recPrP^Sc^ conformers in sPMCA experiments. The results show that BV BH could be seeded by all three protein-only recPrP^Sc^ seeds at high dilutions: (1) Mo protein-only recPrP^Sc^ at 10^−4^ (600 pg/mL PrP^Sc^ seed concentration)(**Fig 2A**, bottom panel) or 10^−5^ (60 pg/mL PrP^Sc^ seed concentration)(**S3 Fig**, bottom row); (2) M109 protein-only recPrP^Sc^ at 10^−4^ (**Fig 2B**, top left panel); and (3) I109 protein-only recPrP^Sc^ at 10^−5^ (**Fig 2B**, middle left panel). In contrast, Mo BH could not be converted by any of the protein-only recPrP^Sc^ seeds, even at the highest concentration tested (0.6 μg/mL)(**Fig 2A**, top panel; **Fig 2B**, right column, top two panels). As expected, we found that M109 cofactor recPrP^Sc^ could seed both Mo BH and BV BH three-round sPMCA reactions at a dilution factor of 10^−5^ (**Fig 2B**, bottom panel). Each sPMCA experiment also contained an unseeded control reaction to control for potential contamination. It has been previously reported that a different protein-only preparation, recPrP amyloid, is able to seed sPMCA reactions at high concentrations [8]. We determined that the minimum concentration of BV recPrP amyloid needed to seed BV BH sPMCA reactions is between 50 μg/mL (**Fig 2C**), which is ~1 million times less potent than protein-only recPrP^Sc^. Moreover, even at a high seeding concentration, the kinetics of PrP^Sc^ formation was slow in recPrP amyloid-seeded reactions, with a sPMCA product becoming first detectable in round 3 (**Fig 2C**, right panel, last lane). Overall, these results show that BV BH is a uniquely susceptible substrate for the propagation of protein-only recPrP^Sc^ seeds, even at high dilutions, in BH sPMCA reactions, and that protein-only recPrP^Sc^ is a highly potent seed, especially compared to protein-only recPrP amyloid.

### Protein-only recPrP^Sc^ seeds are not infectious *in vivo*

**Fig 3:**
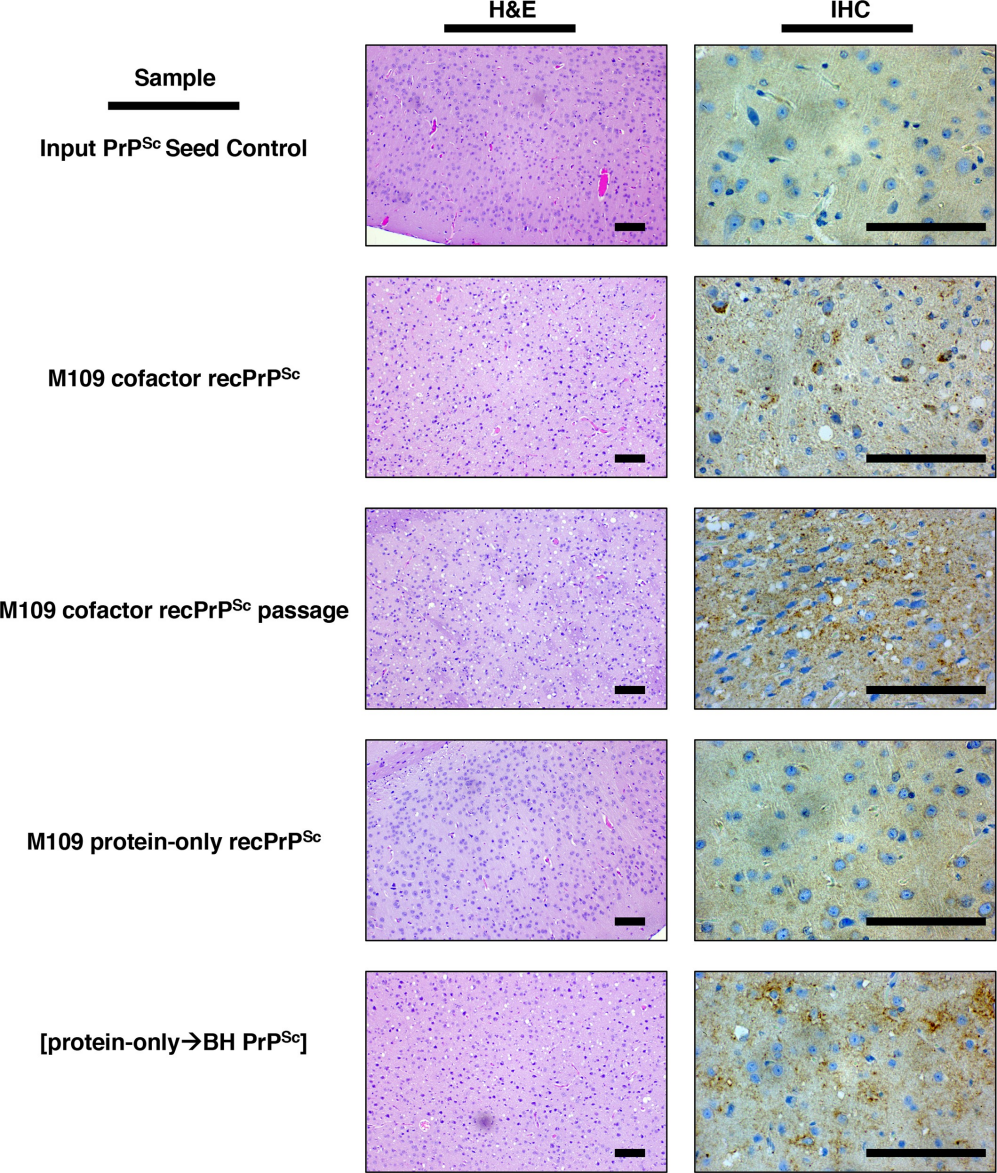
Histopathology of inoculated M109 bank voles. Representative microscopic images of brain sections of M109 bank voles stained with hematoxylin and eosin (H&E) or subjected to immunohistochemistry (IHC) with anti-PrP mAb 27/33, as indicated. Rows from top to bottom: asymptomatic control bank vole sacrificed 410 days after inoculation with a 10^−1^ dilution of the original 6 μg/mL recombinant sPMCA input seed (Mo cofactor recPrP^Sc^) serially diluted 1:10 eighteen times in recombinant sPMCA reaction buffer to demonstrate that there is no remaining infectivity from the input seed; terminally ill bank vole sacrificed 134 days after inoculation with a 10^−1^ dilution of BV M109 cofactor recPrP^Sc^ (final concentration = 0.6 μg/mL); terminally ill bank vole sacrificed 99 days after serial passage of BV M109 cofactor recPrP^Sc^ (10^−1^ dilution of 10% w/v BH); asymptomatic bank vole sacrificed 403 days after inoculation with 10^−1^ dilution BV M109 protein-only recPrP^Sc^ (final concentration = 0.6 μg/mL); and a terminally ill bank vole sacrificed 113 days after inoculation with [protein-only→BH PrP^Sc^] (10^−1^ dilution of round three of the BH sPMCA reaction). The inoculum volume used was 30 μL. Scale bar = 100 μm.

**Fig 4:**
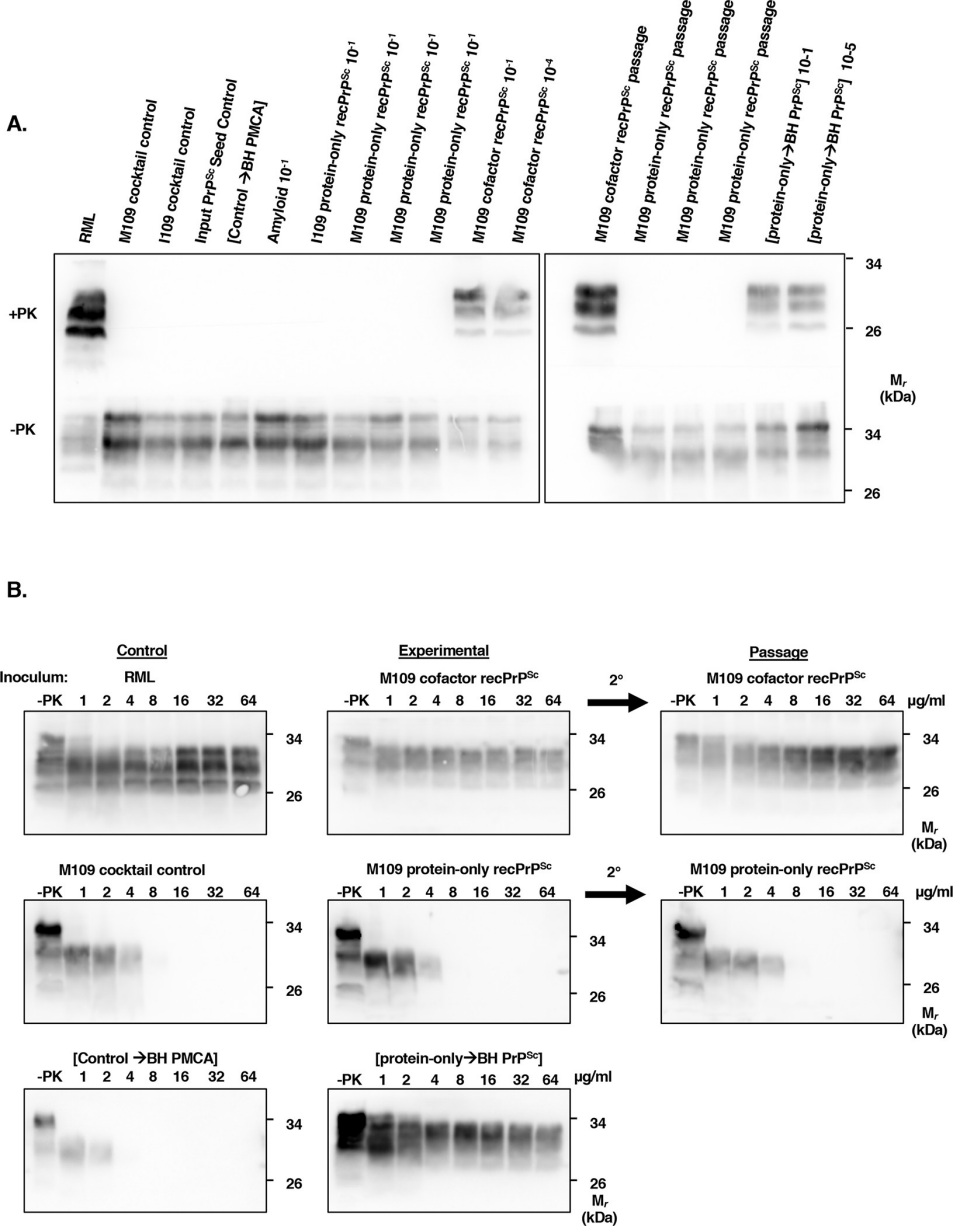
Proteinase K digestion of control and experimentally infected bank voles. **(A)** Western blots probed with anti-PrP mAb 27/33 showing PrP^Sc^ in brain homogenates from M109 bank voles from the indicated control or experimental condition. Top Panel: BH aliquots treated with 64 μg/mL PK (+PK). Bottom panel: BH aliquots that were not subjected to PK digestion (-PK). **(B)** Western blots comparing the protease-resistance levels of PrP^Sc^ in brain homogenates from M109 bank voles from various control or experimental conditions, as indicated. Samples were digested with various concentrations of PK for 1 hr at 37 ^o^C, as indicated. 2° = passage of the experimental sample.

To confirm the *in vivo* susceptibility of bank voles to protein-only PrP^Sc^ conformers as suggested by the sPMCA results, we performed end-point titration bioassays in M109 genotype bank voles. To our surprise, the bioassay results were completely negative despite the ability of protein-only recPrP^Sc^ conformers to potently and rapidly seed BV BH in sPMCA reactions. All bank voles inoculated with a 10^−1^ dilution (30 μL of 0.6 μg/mL PrP^Sc^) of M109 protein-only recPrP^Sc^ remained disease- and symptom-free after 570 days (**Table 1**). Furthermore, voles inoculated with a blind serial passage of brain homogenate prepared from an asymptomatic M109 protein-only recPrP^Sc^-inoculated animal were also asymptomatic after 280 days (**Table 1**). I109 protein-only recPrP^Sc^ and Mo recPrP amyloid also failed to produce disease in bank voles at the 10^−1^ dilution (**Table 1**). The brains of M109 protein-only recPrP^Sc^-inoculated bank voles contained minimal levels of vacuolation and PrP deposition, evident upon histopathological examination (**Fig 3**, fourth row from the top), but lacked protease-resistant PrP, detected by western blot (**Fig 4A**, top row, left panel, samples 2–4 from the right; **Fig 4B**, middle row, left and middle panels). One out of three bank vole brains inoculated with M109 protein-only recPrP^Sc^ showed a very weak positive signal in RT-QuIC (maximum ThT fluorescence: 8%) (**S4 Fig**), but the degree of fibrillization activity did not increase after blind serial passage (**S4 Fig**). Additionally, the brains of blind serial-passaged M109 protein-only recPrP^Sc^-inoculated animals lacked protease-resistant PrP (**Fig 4A**, right panel, samples 2–4 from the left; **Fig 4B**, middle row, right-hand panel). We also inoculated C57BL/6J mice with a 10^−1^ dilution of M109 protein-only recPrP^Sc^. All mice remained disease-free for the duration of their lifespans (**Table 2**), and their brains were histologically normal (**S5 Fig**, bottom row).

**Table 1:** sPMCA using recombinant PrP substrate inoculations in M109 genotype bank voles.

Inoculum	Dilution	n/n_0_	Mean IP (days)	± SEM
Input PrP^Sc^ Seed Control	10^−1^	0/3	>780	
M109 cofactor recPrP^Sc^	10^−1^	13/13	154	± 6
	10^−2^	7/7	193	± 17
	10^−3^	4/5*	239	± 15
	10^−4^	3/3	401	± 46
	10^−5^	0/4^†^	>430	
	10^−6^	0/4'	>450	
M109 protein-only recPrP^Sc^	10^−1^	0/7	>570	
M109 cofactor recPrP^Sc^ passage	10^−1^	4/4	84	± 6
M109 protein-only recPrP^Sc^ blind serial passage	10^−1^	0/3	>280	
I109 protein-only recPrP^Sc^	10^−1^	0/3	>580	
Mouse recPrP Amyloid	10^−1^	0/3	>740	
M109 recPrP + cofactor cocktail control	10^−1^	0/3	>570	
I109 recPrP protein-only cocktail control	10^−1^	0/3	>750	

Bank voles were inoculated with the listed inoculum. recPrP^Sc^ inocula at 10^−1^ dilution have a protein concentration of 0.6 μg/mL. The input PrP^Sc^ seed control is the original 6 μg/mL recombinant sPMCA input seed (Mo cofactor recPrP^Sc^) serially diluted 1:10 eighteen times in recombinant sPMCA reaction buffer to demonstrate that there is no remaining infectivity from the input seed. Cocktail controls contain all the components of a recombinant sPMCA reaction except for the PrP^Sc^ seed. The M109 protein-only recPrP^Sc^ blind serial passage was generated using a 400-day-old asymptomatic BV.

* = 1 vole alive at >400 days, values calculated from animals that became terminally ill.

^†^ = experiment ongoing at >435 days.

‘ = experiment ongoing at >450 days. IP = incubation period until appearance of clinical symptoms. SEM = Standard error of the mean. n/n_0_ = number of animals with clinical symptoms/ total number of animals in the group.

**Table 2:** Inoculations in Mice.

Inoculum	Dilution	n/n_0_	Mean IP (days)	± SEM
Input PrP^Sc^ Seed Control	10^−1^	0/3*	>550	
M109 protein-only recPrP^Sc^	10^−1^	0/3^†^	>570	
M109 cofactor recPrP^Sc^	10^−1^	4/4	436	± 8

C57BL/6J mice were inoculated with the listed inoculum. recPrP^Sc^ inocula at 10^−1^ dilution have a protein concentration of 0.6 μg/mL. The Input PrP^Sc^ seed control is the original 6 μg/mL recombinant sPMCA input seed (Mo cofactor recPrP^Sc^) serially diluted 1:10 eighteen times in recombinant sPMCA reaction buffer to demonstrate that there is no remaining infectivity from the input seed.

* = 1 mouse was sacrificed early at 453 days for incidental health issues. Animal was clinically asymptomatic and diagnostic western blot was negative for PK resistant PrP.

^†^ = 1 mouse was sacrificed early at 412 days for incidental health issues. Animal was clinically asymptomatic. IP = incubation period until appearance of clinical symptoms. SEM = Standard error of the mean. n/n_0_ = number of animals with clinical symptoms/total number of animals in the group.

In contrast, M109 cofactor recPrP^Sc^ caused clinical scrapie in voles at all dilutions from 10^−1^ (100% attack rate, mean incubation period of 154 ± 6 days) to 10^−4^ (100% attack rate, mean incubation period of 401 ± 46 days) (**Table 1**). Upon passage of M109 cofactor recPrP^Sc^, the mean incubation period at a 10^−1^ dilution dropped to 84 ± 6 days (**Table 1**). Clinical symptoms of disease for both primary and second passage included a disappearance of burrowing behavior, an extremely hunched posture, circling, and progressive ataxia. The course of disease lasted approximately two weeks for primary passage, but dropped to several days for second passage. The clinical diagnosis was confirmed by histopathology showing abundant vacuolation and PrP deposition (**Fig 3**, M109 cofactor recPrP^Sc^: second row from the top, M109 cofactor recPrP^Sc^ passage: third row from the top), western blot showing protease-resistant PrP (**Fig 4A**, left panel, right two samples; **Fig 4B**, top row, right two samples), and RT-QuIC showing fibrillization activity in brain homogenates from terminal animals (**S6A Fig**). Importantly, bank voles inoculated with the Input PrP^Sc^ Seed Control sample were clinically asymptomatic (**Table 1**), histologically normal (**Fig 3**, top row), and lacked protease-resistant PrP in their brains (**Fig 4A**, left panel, sample 4 from the left). We also inoculated C57BL/6J mice with a 10^−1^ dilution of M109 cofactor recPrP^Sc^ and observed a 100% attack rate (436 ± 8 days) (**Table 2**), which was confirmed by pathology (**S5 Fig**, middle row). Together, these results show that cofactor recPrP^Sc^ is potently infectious in bank voles and mice, while protein-only recPrP^Sc^ (both M109 and I109) is surprisingly non-infectious in both species, even after blind serial passage.

### Protein-only recPrP^Sc^-seeded brain homogenate is infectious and has the same strain phenotype as cofactor recPrP^Sc^

We were surprised that M109 protein-only recPrP^Sc^ failed to cause scrapie or induce significant levels of PrP^Sc^ accumulation in bank voles, despite its ability to convert BV PrP^C^ to PrP^Sc^ in BH sPMCA quickly and potently. To explore this unexpected result further, we decided to assess the infectivity of the third-round product of BV BH sPMCA reactions seeded by protein-only recPrP^Sc^, which we term [protein-only→BH PrP^Sc^] for simplicity (**S1 Fig**). Therefore, we performed an end-point titration bioassay of [protein-only→BH PrP^Sc^] in bank voles. Remarkably, the results showed that [protein-only→BH PrP^Sc^] is potently infectious in bank voles, causing disease at dilutions from 10^−1^ to 10^−5^ (**Table 3**). At a 10^−1^ dilution, there was a 100% attack rate and a mean incubation period of 113 ± 4 days, calculated as an average of three independent experimental inocula prepared from three separate sPMCA reactions (**Table 3**). Symptomatically, the disease was indistinguishable from that caused by M109 cofactor recPrP^Sc^, but progressed more quickly (4–5-day clinical course). Clinically, we observed a disappearance of burrowing behavior, circling, followed by severe and progressive ataxia, and an extremely hunched posture. PK digestion followed by western blot revealed the accumulation of PrP^Sc^ in the brains of affected animals that was PK resistant at 64 μg/mL, the highest concentration tested (**Fig 4B**, bottom row, middle panel). Pathology revealed the presence of vacuolation and florid PrP deposition in the brains of affected animals (**Fig 3**, bottom row). In contrast, animals inoculated with unseeded BH sPMCA control samples from three separate sPMCA experiments, termed [Control→BH PMCA], remained asymptomatic for at least 320–720 days) (**Table 3**), and an asymptomatic 180-day-old [Control→BH PMCA] vole lacked PK-resistant PrP in its brain (**Fig 4B**, bottom row, left panel). This control confirms the lack of cross-contamination in sPMCA reactions used to generate [protein-only→BH PrP^Sc^].

**Table 3:** [protein-only→BH PrP^Sc^] inoculations into M109 bank voles.

Inoculum	Dilution	n/n_0_	Mean IP (days)	± SEM
[Control→BH PMCA]	10^−1^	0/4	>320	
		0/6	>400	
		0/4	>720	
[protein-only→BH PrP^Sc^]	10^−1^	6/6	104	±3
		3/3	106	0
		3/3	140	±3
	10^−2^	4/4	135	± 5
	10^−3^	1/2'	127	N/A
	10^−4^	4/4	197	± 3
	10^−5^	1/3^†^	250	N/A
	10^−6^	0/4*	>340	

M109 bank voles were inoculated with the listed inoculum. The BH PMCA Control is a 10^−1^ dilution of round three of an unseeded BV BH sPMCA reaction that was sonicated in the same experiment as [protein-only→BH PrP^Sc^] to control for sonicator contamination. Each of the three independent trials of the 10^−1^ inoculation of [protein-only→BH PrP^Sc^] was generated in a separate sPMCA reaction, and had its own [Control→BH PMCA] sample to confirm lack of contamination. [protein-only→BH PrP^Sc^] inocula is a 10^−1^ dilution of round three of a BV BH sPMCA reaction seeded originally with M109 protein-only recPrP^Sc^.

‘ = Group originally contained four animals. Two animals died early of unrelated health issues and were excluded from the data. One animal is ongoing at >280 days.

* = Four animals ongoing at >340 days. N/A = no data available IP = incubation period until appearance of clinical symptoms. SEM = Standard error of the mean. n/n_0_ = number of animals with clinical symptoms/ total number of animals in the group.

^†^ = two ongoing at >335 days.

**Fig 5:**
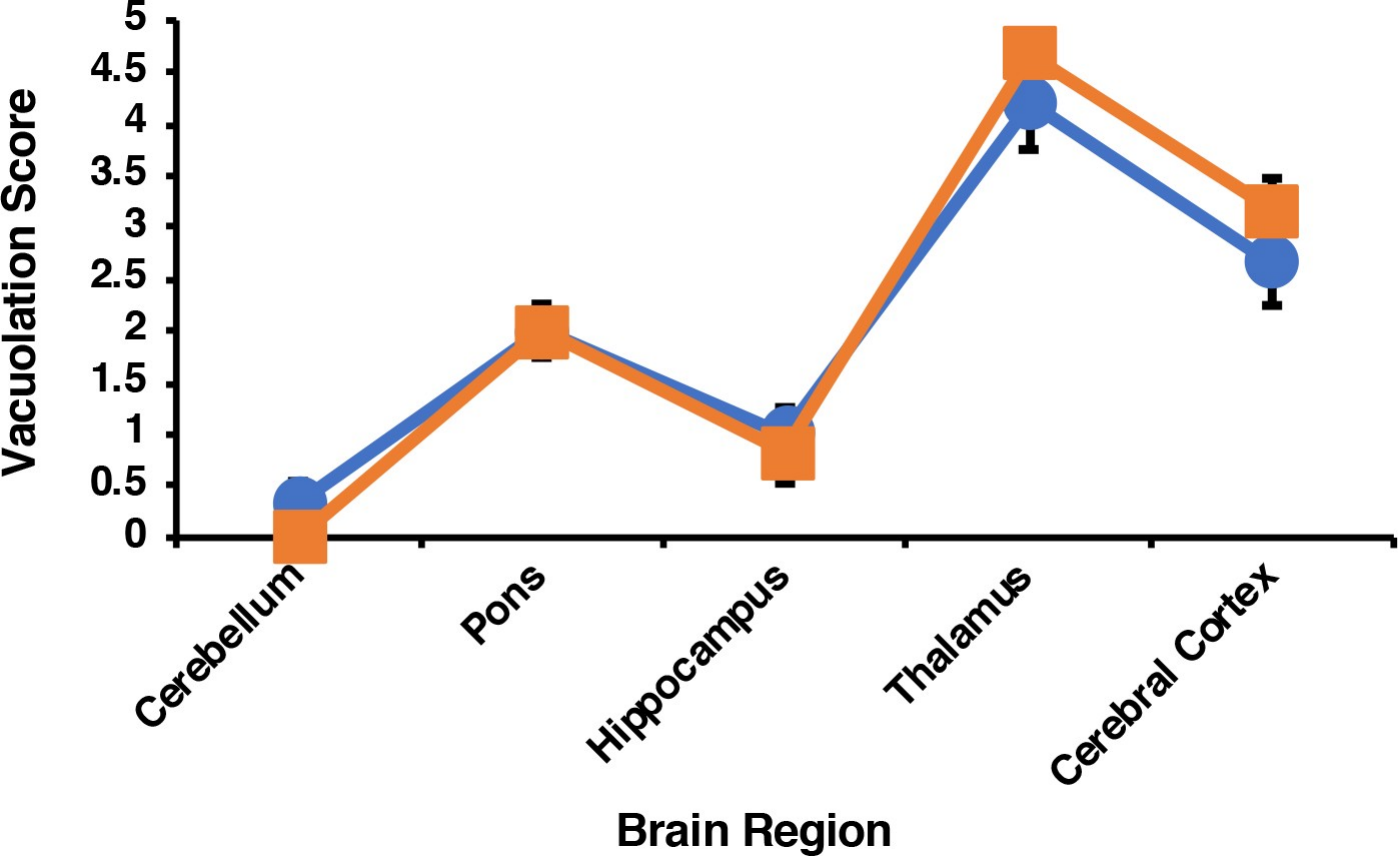
Regional neuropathology of M109 cofactor recPrP^Sc^ and [protein-only→BH PrP^Sc^] infected bank voles. Profiles of vacuolation scores of animals inoculated with either M109 cofactor recPrP^Sc^ (orange squares) or [protein-only→BH PrP^Sc^] (blue circles). Mean values ± SEM are shown. *N* = 6 for all measurements except for [protein-only→BH PrP^Sc^] cerebellum and pons, where *N* = 3.

Given the similarity in clinical symptoms caused by M109 cofactor recPrP^Sc^ and [protein-only→BH PrP^Sc^], we sought to compare the strain properties of these two samples, which share a common provenance (**S1 Fig**). We performed strain typing by examining regional vacuolation in bank voles inoculated with each strain. The two inocula produced a remarkably similar vacuolation pattern (**Fig 5**). Moreover, PrP^Sc^ in the brains of voles infected with either M109 cofactor recPrP^Sc^ or [protein-only→BH PrP^Sc^] displayed similar glycoform ratios and electrophoretic mobility patterns on western blot (**Fig 4**, top row, compare lanes 11 and 12 *vs*. last two lanes), as well as similar degrees of protease resistance (**Fig 4B**, middle column, top *vs*. bottom row). We also used RT-QuIC to compare the seed potency and fibrillization kinetics induced by brain homogenates prepared from animals inoculated with either M109 cofactor recPrP^Sc^ or [protein-only→BH PrP^Sc^] [31]. Both samples showed fibrillization activity at dilutions from 10^−2^ to 10^−8^ (**S6 Fig**). In addition, the time until a positive signal was reached was similar between the two samples: M109 cofactor recPrP^Sc^ BH-seeded brains showed a positive fluorescence signal at a 10^−2^ dilution after 117 minutes, while [protein-only→BH PrP^Sc^] showed a positive fluorescence signal at a 10^−2^ dilution after 80 minutes. Thus, the prions induced by M109 cofactor recPrP^Sc^ and [protein-only→BH PrP^Sc^] cannot be easily discriminated by RT-QuIC. Altogether, the results of these clinical, pathological, and biochemical analyses suggest that M109 cofactor recPrP^Sc^ and [protein-only→BH PrP^Sc^] are very similar or identical strains.

It is important to consider the possibility that the restored infectivity of [protein-only→BH PrP^Sc^] could be due to the contamination from cofactor recPrP^Sc^ seeds; however, this explanation is unlikely for several reasons: (1) all [protein-only→BH PrP^Sc^] and [Control→BH PMCA] inocula were prepared in dedicated, decontaminated sonicators in the absence of any other seeds, including cofactor recPrP^Sc^; (2) special precautions were taken to prevent cross contamination (see Methods) [32]; (3) sentinel [Control→BH PMCA] samples would have detected contaminating cofactor recPrP^Sc^ seeds, as three-round BV BH or Mo BH sPMCA reactions detected 10^−5^ dilutions of M109 cofactor recPrP^Sc^ (**Fig 2**); (4) native PrP^Sc^ accumulated rapidly in the first round of sPMCA (**Fig 1A**, bottom row, protein-only recPrP^Sc^, sPMCA round one), whereas PrP^Sc^ levels due to contamination would be expected to be negligible in the first round and only become detectable in later rounds; (5) identical positive experimental and negative unseeded control biochemical results were obtained in >15 independent experiments; and (6) identical positive experimental and negative control bioassay results were obtained in three independent experiments (**Table 3**).

### Cofactor requirements for protein-only recPrP^Sc^ to convert BV PrP^C^

**Fig 6:**
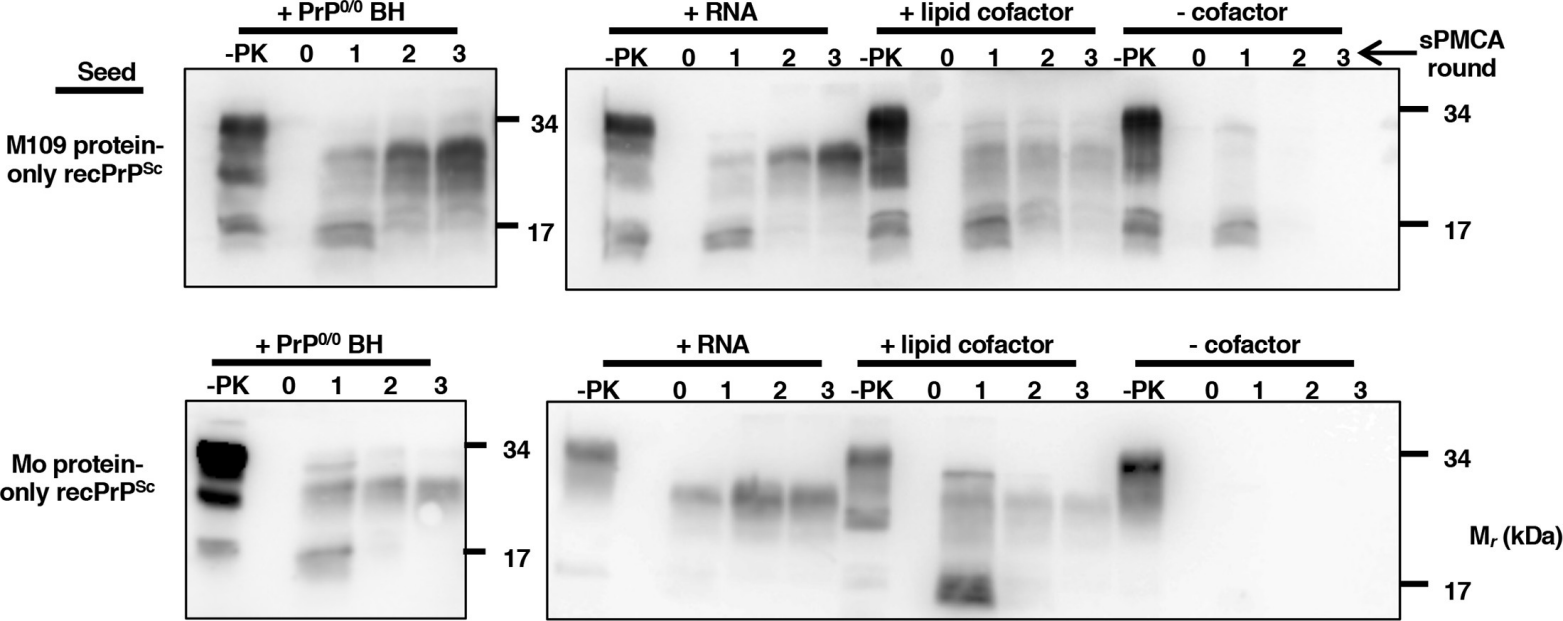
Protein-only recPrP^Sc^ seeds require cofactor molecules to convert immunopurified BV PrP^C^ to PrP^Sc^. Western blots probed with anti-PrP mAb 27/33. Immunopurified M109 BV PrP^C^ substrate was supplemented with, from left to right, PrP^0/0^ BH, RNA, purified lipid cofactor, or PBS and 1% Triton X-100 buffer (-cofactor), as indicated. All of the reconstituted reactions were then seeded with BV M109 protein-only recPrP^Sc^ (top), or Mo protein-only recPrP^Sc^ (bottom), and subjected to three-round sPMCA.

Propagating M109 protein-only recPrP^Sc^ in BV BH sPMCA (to produce [protein-only→BH PrP^Sc^]) led to quantitative recovery of prion infectivity with strain properties indistinguishable from those of M109 cofactor recPrP^Sc^. We sought to determine which biochemical factors were critical for this process. Previous studies have shown cofactor molecules to be essential for the formation of infectious prions *in vitro* [13, 33]. To test whether cofactor molecules are required for M109 protein-only recPrP^Sc^ to convert native BV PrP^C^, we performed reconstituted sPMCA reactions using immunopurified native BV PrP^C^ substrate (**S7 Fig**). Positive control reactions supplemented with BH from PrP^0/0^ mice were able to propagate consistently for three rounds of sPMCA when seeded with either M109 protein-only recPrP^Sc^ or Mo protein-only recPrP^Sc^ (**Fig 6**, top and bottom rows, left-most panels). However, both M109 protein-only recPrP^Sc^ and Mo protein-only recPrP^Sc^ failed to propagate when no source of cofactor was added to the reconstituted sPMCA reaction, indicating that cofactors are essential for protein-only recPrP^Sc^ seeds to convert native BV PrP^C^ (**Fig 6**, top and bottom rows, right-most lanes). Additionally, supplementing reconstituted sPMCA reactions with previously identified, specific cofactor molecules, i.e., either poly(A) RNA molecules or a brain-derived lipid cofactor preparation, facilitated the propagation of both M109 protein-only recPrP^Sc^ and Mo protein-only recPrP^Sc^ (**Fig 6**, top and bottom rows, +RNA, +lipid cofactor columns). Taken together, these results show that cofactors are required for protein-only recPrP^Sc^ seeds to convert native BV PrP^C^, and that either RNA or purified phospholipid can function as the cofactor in this process.

### PrP^C^ post-translational modifications are required to restore prion infectivity

Finally, since native PrP^C^ molecules in BV BH possess a C-terminal glycophosphatidylinositol (GPI) anchor and two N-linked glycans, we also sought to determine whether these post-translational modifications (PTMs) might be necessary for BV PrP^C^ to restore prion infectivity in [protein-only→BH PrP^Sc^]. To do this, we combined bacterially-expressed BV recPrP lacking post-translational modifications together with either RNA or purified phospholipid cofactor molecules as substrate cocktails for sPMCA reactions seeded with M109 protein-only recPrP^Sc^. The results show continued propagation of recPrP^Sc^ for three rounds with either cofactor (**S8 Fig**); however, the MW protease-resistant cores of the sPMCA products appear to be ~16 kDa, which is the same MW as the protease-resistant core of M109 protein-only recPrP^Sc^ seed (**S8 Fig**, compare lanes 2–4 *vs*. lanes 5–10), and smaller than the core of M109 cofactor recPrP^Sc^ (~17 kDa) (**S8 Fig**, last three lanes). These biochemical results suggested that, even in the presence of cofactor molecules, BV recPrP substrate appears to continue propagating the protein-only recPrP^Sc^ confirmation rather than restore the infectious cofactor recPrP^Sc^ conformation. To test this directly, we inoculated the sPMCA products of both recPrP-RNA and recPrP-lipid reactions into bank voles. The results show that neither product is infectious (**Table 4**), confirming that PrP^C^ PTMs do help facilitate the restoration of prion infectivity from protein-only PrP^Sc^.

**Table 4:** Inoculations of M109 protein-only rec. PrP^Sc^-seeded sPMCA reactions using recPrP substrate and cofactor molecules into M109 bank voles.

Inoculum	Dilution	n/n_0_	IP (days)
Unseeded sPMCA control	10^−1^	0/4	>210*
[protein-only→recPrP-lipid PrP^Sc^]	10^−1^	0/3	>210*
[protein-only→recPrP-RNA PrP^Sc^]	10^−1^	0/3	>210*

M109 bank voles were inoculated with the listed inoculum. The unseeded sPMCA control is a 10^−1^ dilution of round three of an unseeded sPMCA reaction with M109 recPrP substrate (without cofactor) that was sonicated in the same experiment as the other listed inocula to control for sonicator contamination. [protein-only→recPrP-lipid PrP^Sc^] is a 10^−1^ dilution of round three of a sPMCA reaction with M109 recPrP substrate supplemented with lipid cofactor seeded originally with M109 protein-only recPrP^Sc^. [protein-only→recPrP-RNA PrP^Sc^] is a 10^−1^ dilution of round three of a sPMCA reaction with M109 recPrP substrate supplemented with poly-A RNA cofactor seeded originally with M109 protein-only recPrP^Sc^.

* incubation ongoing. IP = incubation period until appearance of clinical symptoms. n/n_0_ = number of animals with clinical symptoms/ total number of animals in the group.

## Discussion

We report, for the first time, the *in vitro* restoration of full specific prion infectivity from non-infectious protein-only PrP^Sc^ molecules, rapidly and without strain adaptation. sPMCA propagation of protein-only recPrP^Sc^ in bank vole brain homogenate (BV BH) causes >100,000-fold increase in specific infectivity within three amplification rounds. This restored prion [protein-only→BH PrP^Sc^] displays strain properties that are indistinguishable from M109 cofactor recPrP^Sc^. Several factors appear to be important for the recovery of biological infectivity: (1) the bank vole amino acid sequence; (2) cofactor molecules; (3) post-translational modifications; and (4) sPMCA conditions.

### Native bank vole PrP^C^ is required for restoring prion infectivity from protein-only recPr^PSc^

Our ability to restore biological infectivity from protein-only recPrP^Sc^ was critically dependent upon the remarkable susceptibility of BV PrP^C^ to propagate protein-only recPrP^Sc^ seeds *in vitro*. Notably, BV BH is >100,000-fold more sensitive than Mo BH as substrate for propagating protein-only recPrP^Sc^ seeds *in vitro*, despite the fact that the amino acid sequences of BV PrP^C^ and Mo PrP^C^ are >96% homologous.

Strikingly, BV BH could be potently seeded by Mo protein-only recPrP^Sc^ despite: (1) the inability of Mo protein-only recPrP^Sc^ to seed Mo brain homogenate; and (2) the amino acid differences between seed (which is Mo sequence) and substrate (which is BV sequence). In particular, because native BV PrP^C^, but not native Mo PrP^C^, is susceptible to Mo protein-only recPrP^Sc^, we can be certain that sequence similarity between seed and substrate is not responsible for the remarkable susceptibility of native BV PrP^C^ to protein-only recPrP^Sc^ seeds, in general. This result violates the usual pattern observed for “species barriers” to prion propagation based on primary sequence, in which a perfect sequence match between substrate and seed would be expected to facilitate rather than hinder propagation [34, 35]. Therefore, we can infer that the susceptibility of native BV PrP^C^ substrate to protein-only recPrP^Sc^ seeds is likely due to the primary sequence of BV PrP allowing its structure to be intrinsically more accommodating than PrP sequences to a variety of templates, including protein-only recPrP^Sc^. This interpretation is consistent with the previous observation that recombinant BV PrP substrate is able to propagate PrP^Sc^ seeds in RT-QuIC that were previously undetectable in sPMCA or RT-QuIC using PrP substrates from other species [31]. In general, the amino acid sequence of bank vole PrP^C^ appears to greatly facilitate non-adaptive prion amplification (NAPA) during interspecies transmission[36].

Our data also indicate that N-linked glycans and/or the GPI anchor of BV PrP^C^ are required for the recovery of infectivity from protein-only recPrP^Sc^, since we were unable to restore infectivity using BV recPrP substrate lacking PTMs. Although PTMs are not absolutely required for the formation of prions with high levels of specific infectivity [28, 37, 38], numerous studies have shown that these post-translational modifications can influence PrP folding pathways—sometimes in a strain-dependent manner [39–57]. In our experience, we have never been able to convert purified native PrP^C^ substrate into a protein-resistant conformation in the absence of cofactor molecules. Therefore, we hypothesize that PTMs help prevent native BV PrP^C^ from propagating the protein-only recPrP^Sc^ conformation, which allows it to restore the infectious [protein-only→BH PrP^Sc^] structure instead. On the other hand, recPrP is capable of adopting the protein-only recPrP^Sc^ conformation, and likely prefers to continue propagating this state, even in the presence of cofactor molecules. In other words, continued conversion into protein-only recPrP^Sc^ may serve as a kinetic trap that sequesters recPrP substrate, effectively preventing it from converting into cofactor recPrP^Sc^.

### Cofactor molecules are required for restoring prion infectivity from protein-only recPrP^Sc^

The role played by cofactor molecules in facilitating the formation of infectious prions has been disputed. Using biochemical purification and reconstitution assays, we previously identified single-stranded RNA and PE as essential cofactor molecules for the formation of hamster and mouse prions [13, 33, 58]. Those studies also showed that cofactor molecules are required to produce wild-type prions with significant levels of specific infectivity, and that they restrict the strain properties of synthetic prions [13, 33]. However, using different experimental approaches, others have argued that infectious prions can be formed in the absence of cofactor molecules [10–12].

For instance, while a different I109 protein-only recPrP^Sc^ conformer was reported to be infectious to I109 genotype bank voles [11], its infectivity was characterized by incomplete attack rates (7/9 animals), and the need for an extremely concentrated inoculum (i.e., 5–10 μg/mL for I109 protein-only recPrP^Sc^, which is 10^6^-fold greater than the minimum concentration needed for cofactor recPrP^Sc^) to achieve infection [11]. These observations, combined with the lack of infectivity of I109 protein-only recPrP^Sc^ in our experiments, indicates that the specific infectivity of I109 protein-only recPrP^Sc^ is very low, and may require I109 hosts to be detected. I109 BV PrP^C^ appears be inherently more than prone to misfolding than M109 BV PrP^C^, as transgenic mice overexpressing I109 BV PrP^C^, but not M109 BV PrP^C^, have been shown to develop spontaneous prion disease [59].

A different study reported that amyloid fibrils composed of Mo recPrP 23–144 could cause scrapie in mice [10]; however, an extremely concentrated inoculum (i.e., 100 μg/mL for recPrP 23–144 fibrils, as opposed to a minimal concentration of 60 pg/mL needed for cofactor recPrP^Sc^) was required to induce disease. Although end-point titration experiments were not performed, the large inoculation dose, long incubation period, and large variation in incubation times all suggest that pure recPrP 23–144 fibrils possess very low specific infectivity. Moreover, PrP 23–144 is a truncation mutant linked to Gerstmann-Staüssler Scheinker (GSS) syndrome, a hereditary form of prion disease, and therefore the folding requirements for this mutant may not be shared by wild type PrP^C^. We previously found that other disease-linked PrP mutants can misfold into self-propagating conformers in the absence of cofactor molecules, but that cofactor molecules were ultimately required for those misfolded mutant conformers to seed conversion of wild type PrP^C^ to PrP^Sc^ [60].

Our finding that cofactor molecules are required for protein-only recPrP^Sc^ seeds to convert immunopurified native BV PrP^C^ into PrP^Sc^ indicates that cofactor molecules work together with BV PrP^C^ to restore prion infectivity, and therefore reinforces the concept that cofactor molecules are indeed essential components of infectious wild type prions [16]. It should be noted that although cofactor molecules are required to produce [33], maintain [16, 33], and restore wild-type prions with significant levels of specific infectivity, other studies indicate they are not necessarily sufficient [61–63], demonstrating that specific experimental conditions, i.e., the concentrations and chemical nature of the substrates, physical parameters, etc., must also be optimized to ensure efficient and accurate PrP^Sc^ propagation *in vitro* [64].

### Restoration of full specific infectivity without adaptation *in vitro*

We describe, for the first time, the rapid restoration of fully infectious prions from a protein-only PrP molecule without adaptation (defined here as a slow and inefficient PrP^Sc^ propagation process that ultimately results in a prion strain shift). This has allowed us to dissect the biochemical requirements for the restoration process, as discussed above. Most importantly, it also provides biological evidence that protein-only recPrP^Sc^ must be structurally similar to the infectious conformation of cofactor recPrP^Sc^ and [protein-only→BH PrP^Sc^] prions.

Other investigators have previously produced infectious prions through adaptation by blind serial passage of pure recPrP amyloid in mice [8] and hamsters [9]. It is important to distinguish that these results, although interesting in their own right, must differ fundamentally from those reported here for protein-only recPrP^Sc^ for the following reasons: (1) recPrP amyloid is formed *de novo*, whereas protein-only recPrP^Sc^ is produced by seeded propagation from infectious cofactor recPrP^Sc^; (2) infectious prions induced by recPrP amyloid are formed slowly *in vivo*, whereas [protein-only→BH PrP^Sc^] prions are formed immediately *in vitro*; (3) protein-only recPrP^Sc^ is ~1 million times more potent than recPrP amyloid at seeding formation of BV PrP^Sc^ *in vitro* (compare **Fig 2B** to **2C**); and (4) infectious prions produced by adaptation from recPrP amyloid exhibit novel strain characteristics, whereas the strain characteristics of [protein-only→BH PrP^Sc^] prions are indistinguishable from those of the original cofactor recPrP^Sc^ seed. The strain similarity between [protein-only→BH PrP^Sc^] and cofactor recPrP^Sc^ is particularly striking because [protein-only→BH PrP^Sc^] is composed of native PrP^Sc^ molecules, whereas cofactor recPrP^Sc^ is a recombinant protein lacking PTMs. It should also be noted that the strain similarity between [protein-only→BH PrP^Sc^] and cofactor recPrP^Sc^ cannot be explained by having the same structural constraints imposed by a single purified cofactor [16] because [protein-only→BH PrP^Sc^] was formed using a crude brain homogenate rather than a purified cofactor preparation. We can, therefore, conclude that strain information was successfully maintained and transmitted by the protein-only recPrP^Sc^ structure.

Based on the observations listed above, we infer that recPrP amyloid induces the formation of infectious prions *in vivo* relatively inefficiently and slowly through an adaptation process, most likely by a deformed templating mechanism, as proposed by Baskakov and colleagues [65]. In contrast, the observations suggest that protein-only recPrP^Sc^ likely templates the formation of BV PrP^Sc^ molecules through a relatively high-fidelity, high-efficiency mechanism that requires cofactor molecules similar to the mechanism used during the replication of natural prion strains in the absence of transmission barriers or adaptation. It is unlikely that this mechanism involves selection of a rare, pre-existing protein-only recPrP^Sc^ conformer by cofactor molecules because of the rapidity, potency, and species specificity of the BV BH seeding reactions.

### A unified model of mammalian prion infectivity

Since [protein-only→BH PrP^Sc^] prions with high specific infectivity can be rapidly formed without adaptation from protein-only recPrP^Sc^ seed, we infer that the global structure of protein-only recPrP^Sc^ is likely to resemble those of infectious cofactor recPrP^Sc^ and [protein-only→BH PrP^Sc^] prions, with only small local differences that hinder biological infection. Indeed, deuterium exchange mass spectrometry (DXMS) experiments comparing the structures of cofactor *vs*. protein-only recPrP^Sc^ conformers confirm that the overall structures are similar with subtle differences [17]. An independent comparison between a different pair of infectious versus non-infectious recPrP^Sc^ conformers by Li *et al*. by DXMS yielded similar results[66].

**Fig 7:**
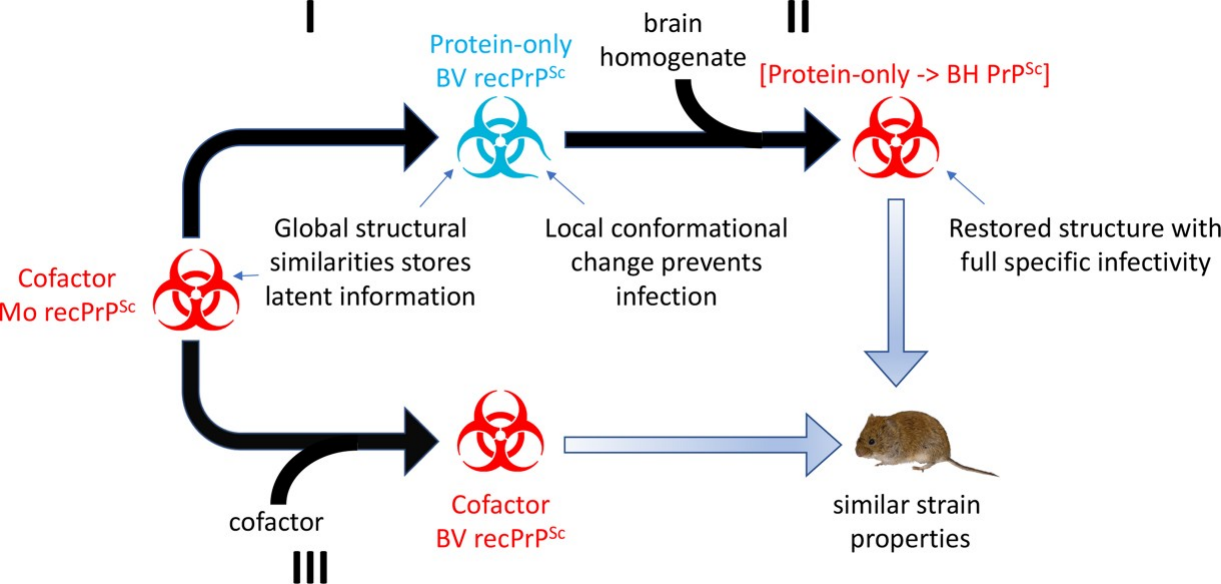
Unified model of mammalian prion infectivity. Proposed model of prion infectivity, in which the global structure of protein-only PrP^Sc^ (formed in reaction I lacking cofactor molecules) can store latent information, but local conformational changes caused by the absence of cofactor abrogates infectivity. The local changes can be repaired by sPMCA in substrate containing BV PrP^C^ and cofactors (reaction II), immediately restoring full specific infectivity. Despite the temporary loss of infectivity, the two-step process (reactions I + II) recovers a prion strain that possesses full specific infectivity and is clinically, biochemically, and pathologically indistinguishable from BV cofactor recPrP^Sc^, in which the specific infectivity of the parental seed was continuously maintained by propagation in the presence of cofactor molecules (reaction III). Non-infectious, protein-only samples are shown in blue, and infectious samples produced with cofactor are shown in red.

Our observation that fully infectious prions with strain properties similar to cofactor recPrP^Sc^ can be rapidly restored from protein-only recPrP^Sc^ suggests a unified model of prion infectivity that reconciles the protein-only hypothesis with the ability of cofactor molecules to increase the specific infectivity of purified native and recombinant prions by many orders of magnitude (**Table 1** and previous work [16, 33]). This model (illustrated in **Fig 7**) proposes that protein-only recPrP^Sc^ molecules are able to maintain and propagate the overall global structure of infectious cofactor recPrP^Sc^, with which it shares a similar provenance (**Fig 7**, reaction I). However, the lack of cofactor molecules causes a subtle conformational change of a local domain that is essential for replication *in vivo* (**Fig 7**, note small anomaly in blue icon). Consistent with this model, DXMS experiments suggest that two domains encompassing residues 91–115 and 144–163 differ in solvent accessibility between cofactor recPrP^Sc^ and protein-only recPrP^Sc^ [17]. Replacing cofactors by sPMCA propagation in BV BH (**Fig 7**, reaction II) repairs the local conformational change [protein-only→BH PrP^Sc^] prions, and thereby restores full prion infectivity. In the end, [protein-only→BH PrP^Sc^] (**Fig 7**, product of sequential reactions I + II) and cofactor recPrP^Sc^ prions (product of reaction II) have similar strain properties because recPrP alone is able to maintain and transmit forward the overall structure of cofactor recPrP^Sc^ prions. Ultimately, high-resolution structural determination of cofactor and protein-only recPrP^Sc^ molecules will be required to confirm this model. In addition, more work is required to determine whether cofactor molecules are also required to propagate infectious prions from other mammalian species, such as cows, deer, and humans.

Our results raise an interesting conundrum: Since protein-only recPrP^Sc^ is a very potent seed for BV BH in sPMCA reactions, and native BV PrP^C^ and cofactor molecules are both present *in vivo*, why isn’t protein-only recPrP^Sc^ directly infectious for bank voles? One possibility is that protein-only recPrP^Sc^ might be degraded *in vivo* more rapidly than infectious conformers. However, as discussed above, protein-only recPrP^Sc^ would be expected to be structurally similar to cofactor recPrP^Sc^, and both of these recombinant conformers lack PTMs such as sialylation, which can influence protein clearance [67, 68]. Therefore, it difficult to envision how cellular prion clearance mechanisms, such as autophagy [69–72] or uptake by resident innate immune cells [73–75], could specifically distinguish between these two conformers. A more likely explanation is that PMCA experimental conditions allow BV PrP^C^ to be more structurally accommodating *in vitro* than *in vivo*. Two specific factors in PMCA experiments that could help make BV PrP^C^ more structurally flexible (and therefore more likely to interact with a structurally imperfect prion template, such as protein-only recPrP^Sc^) are the presence of detergent and cycles of intermittent sonication. Non-ionic detergents, such as Triton X-100, disrupt the plasma membrane to which PrP^C^ is normally attached through its GPI anchor—this disruption could allow PrP^C^ to become more conformationally flexible than when it is anchored to an intact plasma membrane. Likewise, the intense bursts of mechanical energy during sPMCA could cause either PrP^C^ molecules to rapidly sample conformational landscapes that it might not otherwise experience. Other investigators have also observed that sPMCA has the ability propagate PrP^Sc^ conformers that do not infect the corresponding animal hosts or tissues *in vivo* [76–80]. Ultimately, end-point titration bioassay in wild-type animals is the only *bona fide* method to measure specific infectivity [81], and our bioassay data show that protein-only recPrP^Sc^ molecules are non-infectious, whereas [protein-only→BH PrP^Sc^] prions appear to have restored the full specific infectivity and strain properties of cofactor recPrP^Sc^ prions.

In conclusion, we report that prions with high specific infectivity can be rapidly restored from non-infectious protein-only recPrP^Sc^ molecules *in vitro* without adaptation. This provides the first experimental evidence that the conformation of protein-only PrP^Sc^ encodes all the information necessary for infectivity and strain properties, but paradoxically PrP^Sc^ alone is not sufficient for biological infectivity. The unique involvement of cofactor molecules in mammalian prion replication may help explain why, among various self-replicating proteins associated with neurodegenerative diseases in humans, prions are the only ones that are clinically infectious[82].

## Materials and methods

### General sPMCA methods

The general sPMCA experimental method was adapted from Castilla *et al*. [64]. **S1 Fig** diagrams the sPMCA reactions and PrP^Sc^ conformers used in this paper. All PMCA reactions were sonicated in microplate horns at 37°C using a Misonix S-4000 power supply (Qsonica, Newtown, CT) set to power 70 for three rounds. One round of PMCA is equal to 24 hrs. The first round of PMCA was seeded with a volume of PrP^Sc^ equal to 10% of the total reaction volume. To propagate the reaction between PMCA rounds, 10% of the reaction volume was transferred into a new, unseeded, substrate mixture. Due to the sensitivity of sPMCA [32], measures were undertaken to prevent sample contamination. Sample conical tubes were sealed with Parafilm (Bemis Company, Oshkosh, WI) and the sonicator horn was soaked in 100% bleach between experiments to prevent cross-contamination. Sample conical tubes were spun at 500 *x g* for 5 sec to remove liquid off the conical tube lids before propagation and were propagated individually using aerosol resistant pipette tips. The experimenter wore two pairs of gloves and changed the outer layer of gloves when handling a new sample. With each experiment, a sentinel conical tube (a conical tube containing the entire sPMCA reaction mixture but lacking seed) was also placed in the sonicator horn to detect contamination.

### Preparation of cofactor recPrP^Sc^ and protein-only recPrP^Sc^ by sPMCA

Cofactor recPrP^Sc^ and protein-only recPrP^Sc^ were generated by sPMCA based on a previously established protocol [16].

Expression and purification of Mo recPrP 23–230 was performed as previously described [60]. Full-length BV PrP M109 1–255 on pcDNA3.1 and full-length BV PrP I109 1–255 on pcDNA3.1 were used to clone M109 BV recPrP 23–231 and I109 BV recPrP 23–231 onto pET-22b for expression. Site-directed mutagenesis using the Gene Tailor Site Directed Mutagenesis Kit (Invitrogen, Carlsbad, CA) was performed on full-length BV PrP M109 1–255 on pcDNA3.1 and full-length BV PrP I109 1–255 on pcDNA3.1 using the forward primer (GCGCGCCATATGAAGAAGCGGCCAAAG) containing the NdeI cut site and the reverse primer (CGCGCGCTCGAGTCAGGAACTTCTCCC) containing the XhoI cut site. Restriction digest of the PCR products and pET-22b plasmid followed by ligation created the final expression plasmids: BV M109 recPrP 23–231 on pET-22b and BV I109 recPrP 23–231 on pET-22b. These expression plasmids were used to express BV M109 recPrP 23–231 and BV I109 recPrP 23–231 proteins, which were produced and purified as previously described [60].

sPMCA reactions were performed using a previously established protocol with minor modifications [16]. Two-hundred microliter reactions containing 6 μg/mL Mo recPrP 23–230 or BV recPrP 23–231 in conversion buffer (20 mM Tris pH 7.5, 135 mM NaCl, 5 mM EDTA pH 7.5, 0.15% (v/v) Triton X-100) were supplemented with either purified brain-derived phospholipid cofactor [13] for cofactor recPrP^Sc^ propagation, or water for protein-only recPrP^Sc^ propagation. Four BV recPrP samples were created using either BV M109 or BV I109 recPrP, either alone or supplemented with purified brain-derived phospholipid cofactor (**S1** and **S2 Figs**). Each reaction was seeded with Mo cofactor recPrP^Sc^ and propagated for 18 rounds of sPMCA to eliminate the initial input Mo cofactor recPrP^Sc^ seed via serial dilution. Both reactions containing protein alone formed conformers containing protease-resistant cores of ~16 kDa (**S2A Fig**, -cofactor), reminiscent of the MW of the core of Mo protein-only recPrP^Sc^ [16] (**S2B Fig**, bottom panel). The conformers formed from reactions containing protein alone were termed I109 protein-only recPrP^Sc^ and M109 protein-only recPrP^Sc^. The reaction containing M109 recPrP and brain-derived lipid cofactor formed a stably propagating conformer with a protease-resistant core of ~17 kDa (**S2A Fig**, +cofactor), slightly lower than the MW of the core of Mo cofactor recPrP^Sc^ [16] (**S2B Fig**), and was termed M109 cofactor recPrP^Sc^. However, the MW of recPrP^Sc^ produced in reactions containing I109 recPrP plus brain-derived lipid cofactor substrate consistently shifted to ~16 kDa after 2–3 rounds of sPMCA (**S2C Fig**). Since this conformer migrated at the same MW as the protein-only recPrP^Sc^ conformers, we decided not to include it in further experiments. All sPMCA reactions were sonicated with 15-sec pulses every 30 min.

### sPMCA with brain homogenate substrate

Bank vole brains were harvested from animals with M109 genotype perfused with PBS plus 5 mM EDTA. A 10% (w/v) perfused BH substrate was prepared in PBS, 1% (v/v) Triton X-100, 5 mM EDTA, and cOmplete Mini Protease Inhibitors (Roche, Basel, Switzerland). For sPMCA titrations, 10-fold serial dilutions of cofactor recPrP^Sc^ or protein-only recPrP^Sc^ seeds were created in conversion buffer [20 mM Tris pH 7.5, 135 mM NaCl, 5 mM EDTA pH 7.5, 0.15% (v/v) Triton X-100]. Reactions were sonicated with 20-sec pulses every 30 min.

### Immunopurification of PrP^C^ from brain tissue

PrP^C^ was immunopurified from BV (genotype M109) brains based on a previously established protocol [33]. Using an electric potter homogenizer, 12 g of BV brains were homogenized in 80 mL Buffer A (20 mM MOPS pH 7.0, 150 mM NaCl) with cOmplete Protease Inhibitor Cocktail tablets (Roche). The resulting homogenate was centrifuged at 3200 *x g* for 25 min at 4°C. The supernatant was discarded, and the pellets were resuspended to a volume of 40 mL by Dounce homogenizing in Buffer A, 1% (w/v) sodium deoxycholate, 1% (v/v) Triton X-100. The homogenate was incubated on ice for 30 min to solubilize PrP^C^, then centrifuged at 100,000 *x g* for 40 min at 4°C.

The solubilized supernatant was placed into a 50-mL conical tube with 1 mL of Protein A agarose (Pierce, Rockford, IL) and end-over-end rotated for 30 min at 4°C as a pre-clear step. Next, the supernatant/Protein A mixture was poured through an Econo-Pac (Bio-Rad, Hercules, CA) column and the flow-thru was collected as the pre-cleared load.

The pre-cleared load was passed over a column packed with 2 mL of Protein A Agarose resin (Pierce) cross-linked to 6D11 mAb that was pre-equilibrated with Buffer A, 1% (w/v) sodium deoxycholate, 1% (v/v) Triton X-100 at a flow rate of 0.75 mL/min. The column was washed with 36 mL of Wash Buffer 1 [20 mM Tris pH 8.0, 1% (v/v) Triton X-100, 500 mM NaCl, 5 mM EDTA], followed by 24 mL of Wash Buffer 2 [Buffer A, 0.5% (v/v) Triton X-100] at a flow rate of 1.0 mL/min. A 50-mL conical tube containing 900 μL of Neutralization Buffer [1M Tris pH 9.0, 5% (v/v) Triton X-100, 1.4 M NaCl] was placed beneath the column. The column was manually eluted using a syringe filled with Elution Buffer (0.1 M glycine pH 2.5, 100 mM NaCl) until a volume of 15 mL was reached.

The eluate was brought to 50 mL with SP Equilibration/Wash Buffer [20 mM MES pH 6.4, 0.15 M NaCl, 0.5% (v/v) Triton X-100] and applied slowly to a 1.5-mL SP Sepharose (Sigma Aldrich, St. Louis, MO) ion-exchange column that was pre-equilibrated with 10 column volumes of SP Equilibration/Wash Buffer. The column was washed with 15 mL of SP Equilibration/Wash Buffer and eluted with 5 mL of SP Elution Buffer [20 mM MOPS pH 7.5, 0.50 M NaCl, 1% (v/v) Triton X-100] containing cOmplete EDTA-free Protease Inhibitor Cocktail tablets (Roche). The eluate was dialyzed in 3500 MWCO Slide-a-Lyzer (Pierce) cassettes overnight against 4 L of Exchange Buffer [20 mM MOPS pH 7.5, 150 mM NaCl, 0.5% (v/v) Triton X-100].

### sPMCA using immunopurified PrP^C^

Reconstituted sPMCA experiments were adapted from Piro *et al*. [83]. Briefly, 150-μL reactions containing 20 μg/mL immunopurified BV M109 PrP^C^ in conversion buffer (20 mM MOPS pH 7.0, 0.075% Triton X-100, 50 mM imidazole pH 7.0, 5 mM EDTA pH 7.5, 0.1 M NaCl) were supplemented with either 45 μL of 10% (w/v in PBS) PrnP^0/0^ BH, purified brain-derived phospholipid cofactor [13], PBS and 1% (v/v) Triton X-100, or 60 μg/mL polyadenylic acid potassium salt (Sigma Aldrich). Reactions were sonicated with 20-sec pulses every 30 min.

### Detection of PrP^Sc^ in sPMCA reactions

Formation of PrP^Sc^ was monitored by digestion of sPMCA samples with Proteinase K (PK) (Roche) and western blotting. Samples were digested with 64 μg/mL PK at 37°C with shaking at 750 r.p.m. Samples from sPMCA reactions using recPrP as the substrate were treated for 30 min, while samples using BH or immunopurified PrP^C^ as the substrate were treated for 60 min. Digestion reactions were quenched by adding SDS-PAGE loading buffer and heating to 95°C for 15 min. SDS-PAGE and western blotting were performed as described previously [83] using mAb 27/33 (epitope = 136–158 mouse numbering). Then, 20 μL of a sPMCA reaction was subjected to PK digestion. The minus (-) PK lane shown in each western blot figure is used to determine the conversion efficiency of a sPMCA reaction. The amount of PrP^C^ in the original substrate relative to the amount that was converted to PrP^Sc^ during one round of PMCA. For reactions using recPrP as the substrate, the minus PK lane contains the same volume (20 μL) of a sPMCA reaction as a PK-digested sample. For reactions using BH or immunopurified PrP^C^ as the substrate, the minus PK lane contains one-tenth (2 μL) of the volume used in the PK-digested samples.

### Amyloid fiber preparation

Amyloid fibers were generated as previously described [84]. Briefly, a 3.0-mg/mL stock of recPrP was made by adding 6.0 M GdnHCl to the lyophilized protein. A 1.5-mL conical tube containing a 600-μL reaction volume (2 M GdnHCl, 50 mM MES buffer, pH 6.0, 10 mM thiourea, and 250 μg of recPrP) was incubated at 37°C with continuous shaking at 1700 r.p.m. for 24 h. Fibers were centrifuged at 100,000 x *g* and then washed with 10 mM NaAc pH 5.0 twice and stored at 4°C.

### Animal inoculations, diagnosis, and neuropathology

Intracerebral inoculation and diagnosis of prion disease were performed as described [83] with the following modifications: PMCA mixtures and products were diluted 1:10 into PBS plus 1% (w/v) bovine serum albumin before inoculation. Brain homogenate samples (10% w/v in PBS) were spun for 30 sec at 200 *x g* to remove nuclear debris, and the supernatant was collected and used as the inoculum. The inoculum volume used was 30 μL. Bank voles with the M109 genotype were bred from a colony originally established at the Istituto Superiore di Sanità (Rome, Italy), and inoculated between 4–6 weeks of age. Neuropathology was performed as previously described [13], using primary mAb 27/33 at a 1:1000 dilution and a Biocare Mouse on Mouse Horseradish Peroxidase Polymer (Biocare Medical, Pacheco, CA) for the immunohistochemical detection of PrP.

### Ethics statement

The Guide for the Care and Use of Laboratory Animals of the National Research Council was strictly followed for all animal experiments. All experiments involving voles and mice in this study were conducted in accordance with protocol supa.su.1 as reviewed and approved by Dartmouth College’s Institutional Animal Care and Use Committee, operating under the regulations/guidelines of the NIH Office of Laboratory Animal Welfare (assurance number A3259-01) and the United States Department of Agriculture.

### Proteinase K digestion and detection of PrP^Sc^ in experimentally infected brains

Formation of PrP^Sc^ was monitored by digestion of BHs [10% (w/v) in PBS] with PK followed by western blotting. Samples were digested in a reaction containing 64 μg/mL PK (unless otherwise specified), 2% (v/v) Tween-20 (Fisher Scientific, Hampton, NH), 2% (v/v) NP-40 (Fisher Scientific, Hampton, NH), and 2% (w/v) n-Octyl-β-D-Glucopyranoside (Anatrace, Maumee, OH) at 37°C with shaking at 750 r.p.m. for 1 hr. Digestions were quenched by adding SDS-PAGE loading buffer and heating to 95°C for 15 min. SDS-PAGE and western blotting were performed as described previously [83] using mAb 27/33. Twenty microliters of a brain homogenate were subjected to PK digestion. The minus PK lane is used to determine the fraction of PrP that has been converted to PrP^Sc^ in the brain. The minus PK lane contains the same volume (20 μL) of BH as a PK-digested sample.

### Real-time quaking induced conversion assay

RT-QuIC reactions were carried out as described previously [31], with the following modifications. Lyophilized BV M109 recPrP was resuspended in 10 mM sodium phosphate (pH 5.8) to a concentration of 0.5 mg/mL. The resuspended protein was filtered through a 0.22-μm syringe-driven filter, and the concentration was adjusted using 10 mM sodium phosphate (pH 5.8) to a concentration of 0.3 mg/mL. The resuspended protein was then diluted in a reaction buffer (10 mM sodium phosphate buffer pH 7.4, 300 mM NaCl, 10 μM ThT, 1 mM EDTA, and 0.001% SDS) to a final concentration of 0.1 mg/mL. Ninety-eight microliters of this reaction mixture was added to each well of a black-walled 96-well plate with a clear bottom with 2 μL of seed. Ten-fold serial dilutions of seeds were created in PBS and 0.025% (v/v) SDS. The plate was sealed and incubated at 42°C with 90-sec intervals of orbital shaking at 920 r.p.m. followed by 90 sec of rest in a FilterMax F5 Multi-Mode Microplate Reader (Molecular Devices, San Jose, CA). ThT fluorescence measurements (430 +/- 35-nm excitation and 485 +/- 20-nm emission) were taken every three min. Experimental samples were run in technical triplicate. Data analysis was performed as described previously [85], except the mean baseline relative fluorescence units were calculated over a one-hr period.

## Supporting information

S1 Fig Schematic diagram of *in vitro* PrP^Sc^ molecule generation. (PDF)

S2 Fig Generation of BV recPrP^Sc^ conformers. (PDF)

S3 Fig Deepest titration of Mo protein-only recPrP^Sc^ into BV BH observed. (PDF)

S4 Fig RT-QuIC seeding activity of BHs from control bank voles and M109 protein-only recPrP^Sc^ and blind serial-passage-inoculated animals. (PDF)

S5 Fig Histopathology of inoculated mice. (PDF)

S6 Fig RT-QuIC seeding activity of BHs from [protein-only→BH PrP^Sc^]-inoculated bank voles BH and M109 cofactor recPrP^Sc^-inoculated bank voles. (PDF)

S7 Fig Silver stain analysis of immunopurified M109 BV PrP^C^ substrate. (PDF)

S8 Fig Addition of cofactors does not alter the MW of propagating M109 protein-only recPrP^Sc^. (PDF)
