# Supplementary material for: Full restoration of specific infectivity and strain properties from pure mammalian prion protein
Source: PLoS Pathog. 2019 Mar 25;15(3):e1007662. doi: 10.1371/journal.ppat.1007662 (PMC6448948; doi:10.1371/journal.ppat.1007662)
Supplement: S1 Fig — (PDF) [file ppat.1007662.s001.pdf]

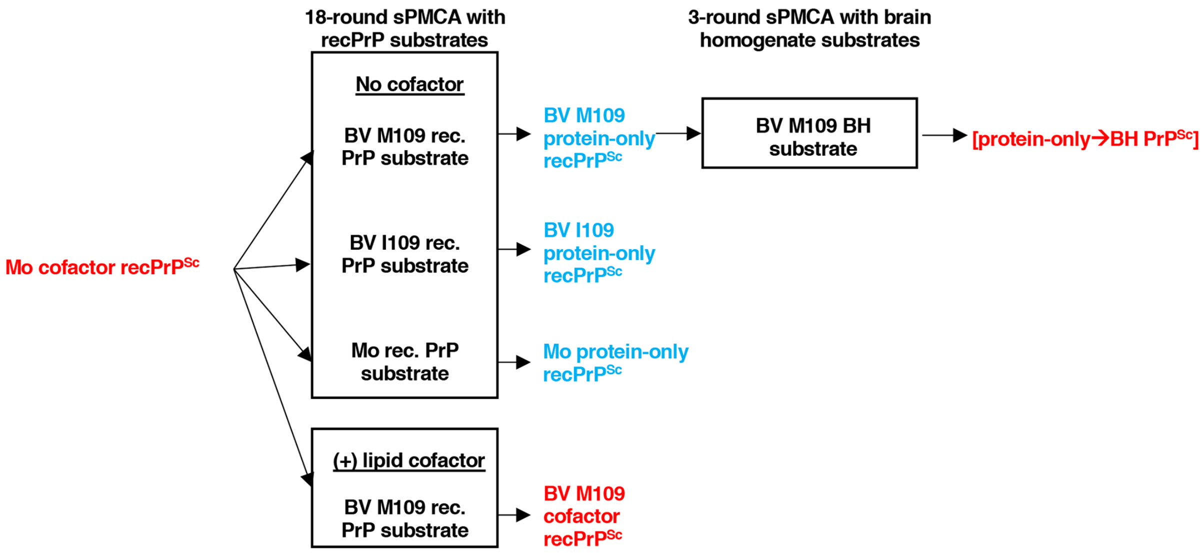

S1 Fig: Schematic diagram of *in vitro* PrP^{Sc} molecule generation.

All strains were originally created using Mo cofactor $recPrP^{Sc}$. Mo protein-only $recPrP^{Sc}$, BV M109 cofactor $recPrP^{Sc}$, BV M109 protein-only $recPrP^{Sc}$, BV I109 protein-only $recPrP^{Sc}$ were created by adapting Mo cofactor $recPrP^{Sc}$ in sPMCA using recombinant PrP substrate for 18 rounds under the indicated conditions to remove the initial Mo cofactor $recPrP^{Sc}$ seed. [protein-only→BH PrP^{Sc}] was created by propagating BV M109 protein-only $recPrP^{Sc}$ for three rounds in BV BH sPMCA. Protein-only samples are indicated in blue font, and samples produced with cofactor are indicated in red font.
