# Supplementary material for: Full restoration of specific infectivity and strain properties from pure mammalian prion protein
Source: PLoS Pathog. 2019 Mar 25;15(3):e1007662. doi: 10.1371/journal.ppat.1007662 (PMC6448948; doi:10.1371/journal.ppat.1007662)
Supplement: S2 Fig — (PDF) [file ppat.1007662.s002.pdf]

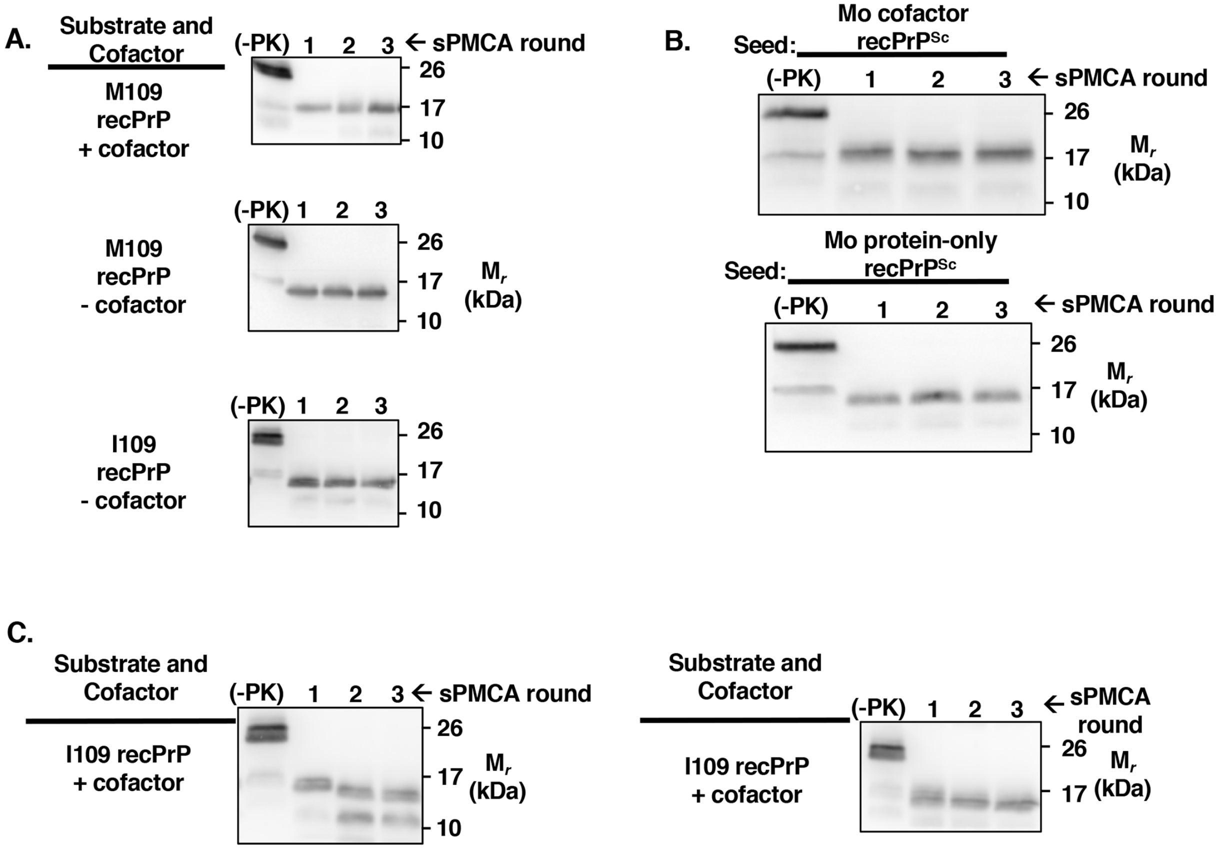

S2 Fig: Generation of BV recPrP^{Sc} conformers. Western blots probed with anti-PrP mAb 27/33 (epitope = 136–158 mouse numbering). **(A)** Generation of recombinant BV PrP^{Sc} conformers. Thee-round sPMCA reactions using either M109 BV recPrP or I109 BV recPrP substrate were initially seeded with Mo cofactor recPrP^{Sc}, and propagated in mixtures with or without brain-derived lipid cofactor, as indicated. **(B)** Generation of recombinant Mo PrP^{Sc} conformers. Three-round sPMCA reactions using Mo recPrP substrate were either seeded with Mo cofactor recPrP^{Sc} and propagated in the presence of lipid cofactor (top panel) or seeded with Mo protein-only recPrP^{Sc} and propagated in the absence of lipid cofactor (bottom panel). **(C)** BV I109 recPrP^{Sc} fails to stably maintain higher-MW weight band after PK digestion. Two independent three-round sPMCA using BV I109 recPrP substrate and supplemented with lipid cofactor were seeded with Mo cofactor recPrP^{Sc}.
