# Supplementary material for: Full restoration of specific infectivity and strain properties from pure mammalian prion protein
Source: PLoS Pathog. 2019 Mar 25;15(3):e1007662. doi: 10.1371/journal.ppat.1007662 (PMC6448948; doi:10.1371/journal.ppat.1007662)
Supplement: S3 Fig — (PDF) [file ppat.1007662.s003.pdf]

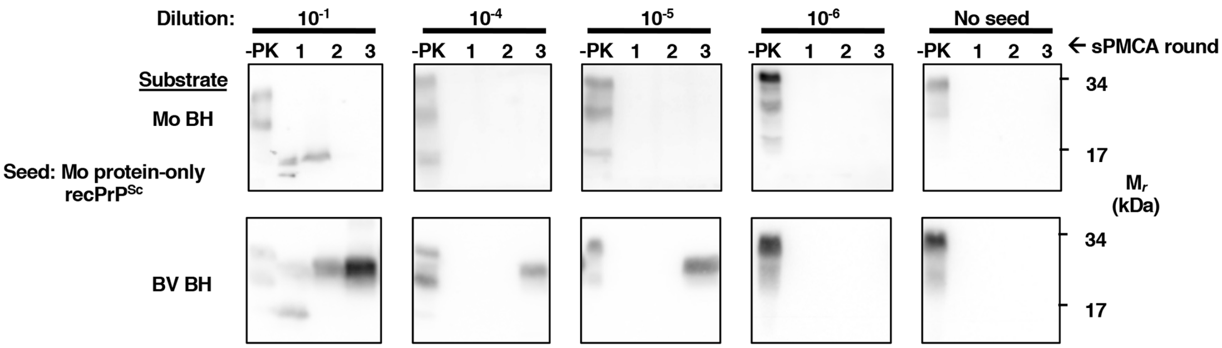

S3 Fig: Deepest titration of Mo protein-only recPrP^{Sc} into BV BH observed. Western blots probed with anti-PrP mAb 27/33 showing three-round BH sPMCA reactions with either Mo BH (top row) or BV BH (bottom row) substrates. Reactions were seeded with 10-fold serial dilutions of Mo protein-only recPrP^{Sc}. The 10^{-1} reaction was seeded with 6 µg/mL of Mo protein-only recPrP^{Sc} for a final reaction concentration of 0.6 µg/mL of seed. This figure is a replicate of the experiment shown in Fig. 2A, showing the highest level of sensitivity (10^{-5} dilution) that we observed for BV BH substrate seeded with protein-only recPrP^{Sc}.

.
