# Supplementary material for: Full restoration of specific infectivity and strain properties from pure mammalian prion protein
Source: PLoS Pathog. 2019 Mar 25;15(3):e1007662. doi: 10.1371/journal.ppat.1007662 (PMC6448948; doi:10.1371/journal.ppat.1007662)
Supplement: S4 Fig — (PDF) [file ppat.1007662.s004.pdf]

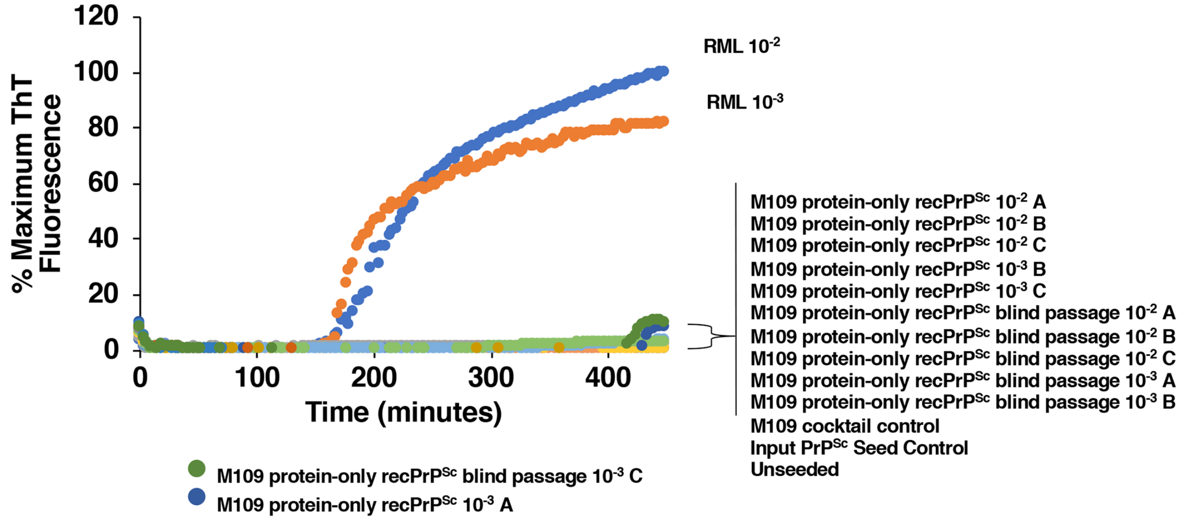

S4 Fig: RT-QuIC seeding activity of BHs from control bank voles and M109 protein-only recPrP^{Sc} and blind serial-passage-inoculated animals. RT-QuIC reactions were seeded with the indicated dilution of 10% brain homogenates from various samples, as indicated. A, B, and C samples are different brains from the same inoculation group (i.e., biological replicates). Data points are the average of technical triplicate samples. Samples were tested simultaneously in the same sealed 96-well plate. The data presented in this figure are representative of two independent experiments.
