# Supplementary material for: Full restoration of specific infectivity and strain properties from pure mammalian prion protein
Source: PLoS Pathog. 2019 Mar 25;15(3):e1007662. doi: 10.1371/journal.ppat.1007662 (PMC6448948; doi:10.1371/journal.ppat.1007662)
Supplement: S5 Fig — (PDF) [file ppat.1007662.s005.pdf]

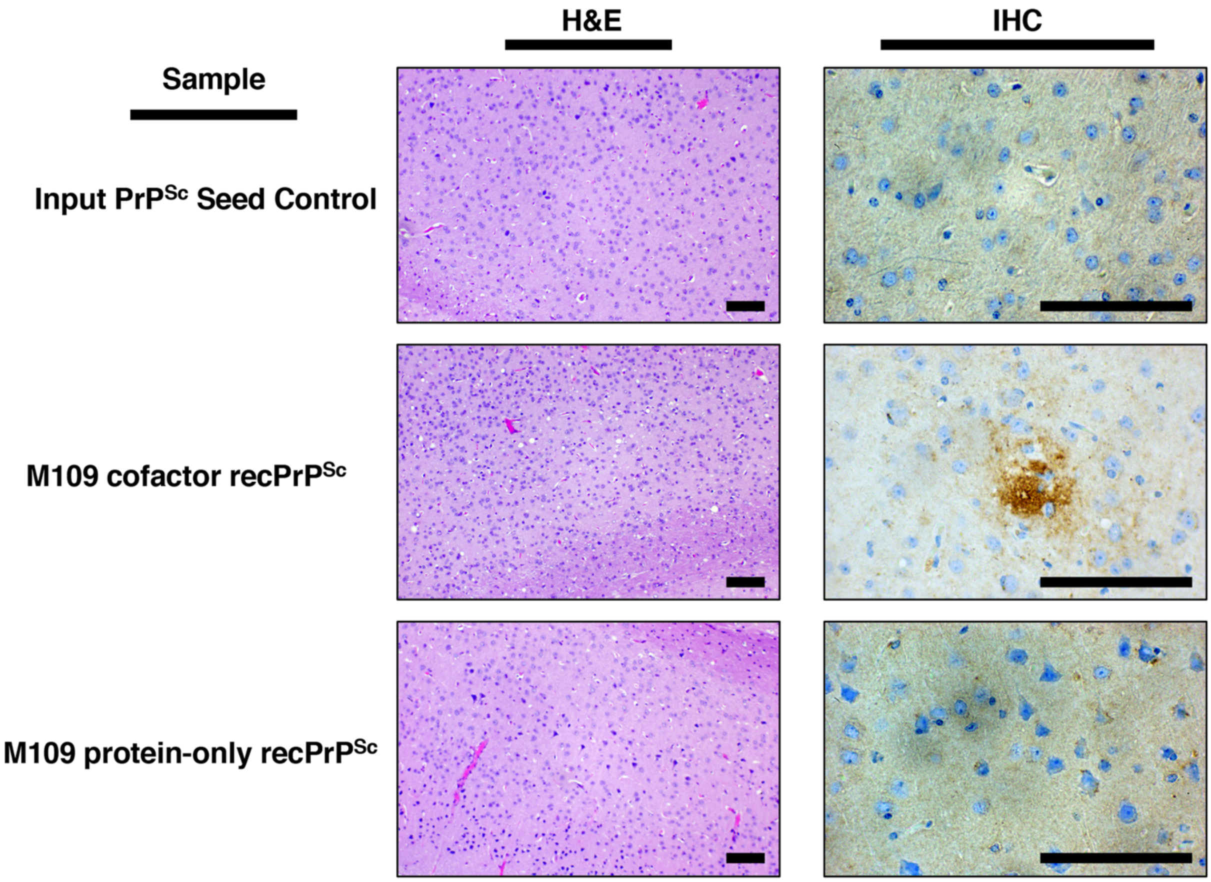

S5 Fig: Histopathology of inoculated mice. Representative microscopic images of brain sections of C57BL/6J mice stained with hematoxylin and Eosin (H&E) or subjected to immunohistochemistry (IHC) with primary anti-PrP mAb 27/33, as indicated. Rows from top to bottom: asymptomatic control mouse sacrificed 558 days after inoculation with a 10^{-1} dilution of the original 6 µg/mL recPrP^{Sc} input seed (Mo cofactor recPrP^{Sc}) serially diluted 1:10 18 times in recombinant sPMCA reaction buffer to demonstrate that there is no remaining infectivity from the input seed, terminally-ill mouse sacrificed 447 days after inoculation with a 10^{-1} dilution BV M109 cofactor recPrP^{Sc} (final concentration = 0.6 µg/mL), asymptomatic mouse sacrificed 574 days after inoculation with a 10^{-1} dilution BV M109 protein-only recPrP^{Sc} (final concentration = 0.6 µg/mL). The inoculum volume used was 30 µL. Scale bar = 100 µm.
