# Supplementary material for: Full restoration of specific infectivity and strain properties from pure mammalian prion protein
Source: PLoS Pathog. 2019 Mar 25;15(3):e1007662. doi: 10.1371/journal.ppat.1007662 (PMC6448948; doi:10.1371/journal.ppat.1007662)
Supplement: S6 Fig — (PDF) [file ppat.1007662.s006.pdf]

A.

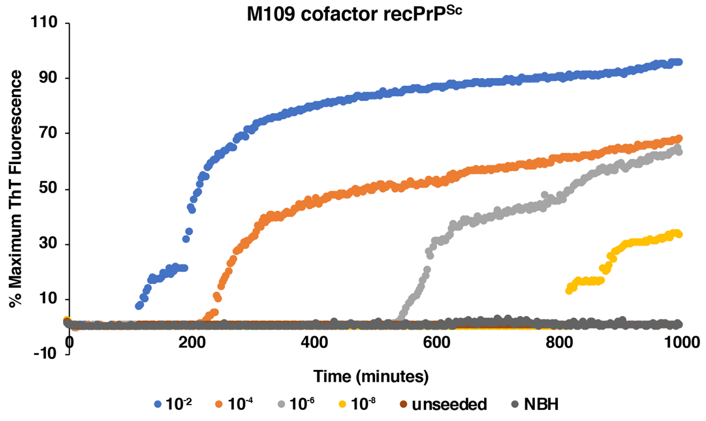

B.

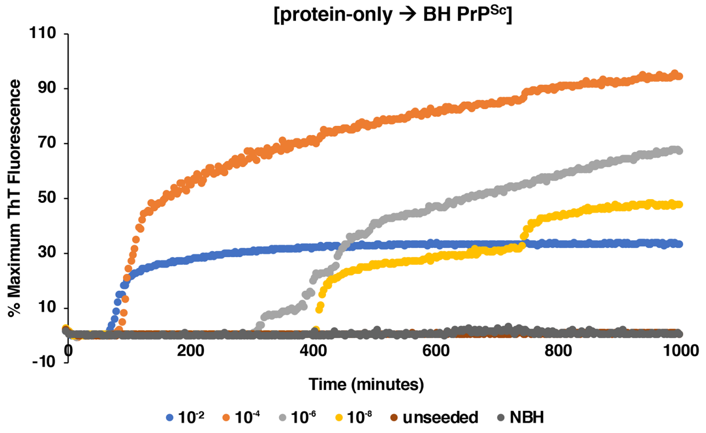

S6 Fig: RT-QuIC seeding activity of BHs from [protein-only→BH PrP^{Sc}]-inoculated bank voles BH and M109 cofactor recPrP^{Sc}-inoculated bank voles. RT-QuIC reactions were seeded with the indicated dilutions of 10% BH from bank voles inoculated with **(A)** M109 cofactor recPrP^{Sc} or **(B)** [protein-only→BH PrP^{Sc}]. NBH = BH from an uninoculated M109 BV. Data points are the average of technical triplicate samples, and data are representative of results obtained from three different brains per experimental condition. Samples were tested simultaneously in the same sealed 96-well plate. The same NBH and unseeded reaction controls are displayed for each graph.
