# Supplementary material for: Full restoration of specific infectivity and strain properties from pure mammalian prion protein
Source: PLoS Pathog. 2019 Mar 25;15(3):e1007662. doi: 10.1371/journal.ppat.1007662 (PMC6448948; doi:10.1371/journal.ppat.1007662)
Supplement: S7 Fig — (PDF) [file ppat.1007662.s007.pdf]

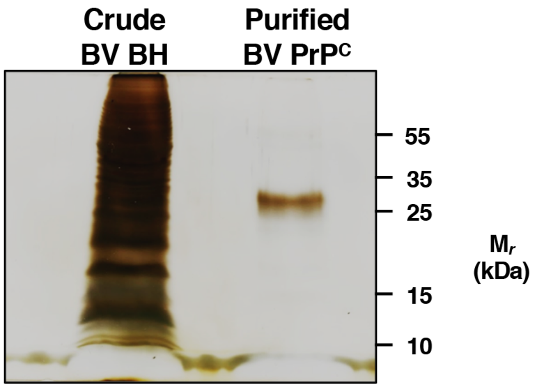

S7 Fig: Silver stain analysis of immunopurified M109 BV PrP^C substrate. Silver-stained 12% SDS-PAGE gel showing crude, detergent solubilized BV M109 BH (left lane) and immunopurified BV PrP^C (right lane).
