# Supplementary material for: Full restoration of specific infectivity and strain properties from pure mammalian prion protein
Source: PLoS Pathog. 2019 Mar 25;15(3):e1007662. doi: 10.1371/journal.ppat.1007662 (PMC6448948; doi:10.1371/journal.ppat.1007662)
Supplement: S8 Fig — (PDF) [file ppat.1007662.s008.pdf]

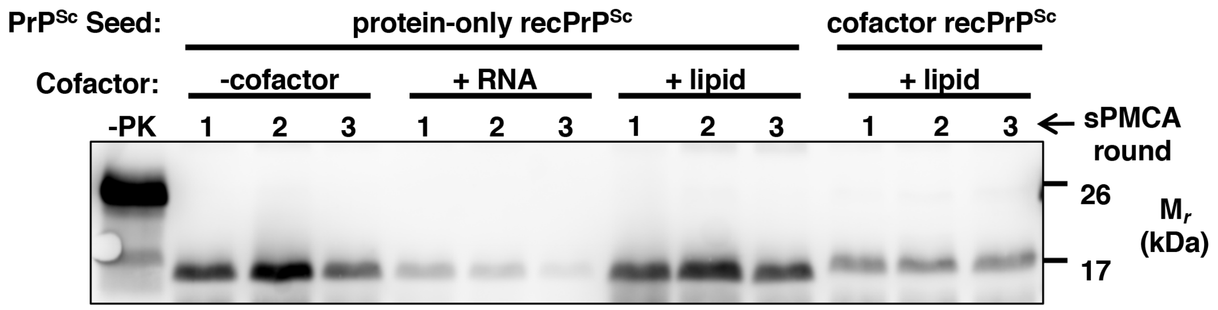

S8 Fig: Addition of cofactors does not alter the MW of propagating M109 protein-only recPrP^{Sc}. Western blots probed with anti-PrP mAb 27/33 showing three-round sPMCA using recombinant PrP substrate reactions using BV M109 recPrP as the substrate alone (-cofactor) or supplemented with RNA (+RNA) or purified brain-derived phospholipid cofactor (+lipid). Reactions were seeded with either M109 protein-only recPrP^{Sc} or M109 cofactor recPrP^{Sc}, as indicated.
